# CBR-Based Skeleton Optimization for the Volumetric Design of Porous Asphalt Mixtures

**DOI:** 10.3390/ma19183984

**Published:** 2026-09-19

**Authors:** Jixing Jie, Xiaoquan Xiao, Qing Wang, Jian Li, Yuling Zhang, Bo Chen

**Affiliations:** 1 School of Civil Engineering & Transportation, South China University of Technology, Guangzhou 510630, China; 202320210190@mail.scut.edu.cn (J.J.); 202611090518@mail.scut.edu.cn (Y.Z.); 2 Guangzhou Xiaoning Road Engineering & Technology Research Office Co., Ltd., Guangzhou 510630, China; oyeoye@21cn.com (X.X.); 1112616023@e.gzhu.edu.cn (J.L.); 3 Guangdong Guanyue Road and Bridge Co., Ltd., Guangzhou 511400, China; wangqinggylq@163.com; 4 School of Civil and Transportation Engineering, Guangzhou University, Guangzhou 510006, China; 5 School of Civil and Transportation Engineering, Foshan University, Foshan 528225, China

**Keywords:** porous asphalt concrete, California Bearing Ratio, coarse aggregate void filling, aggregate skeleton, gyratory compaction, volumetric design, sound absorption

## Abstract

**Highlights:**

- *CBR*_5.0_ enables front-end, mechanics-based selection of PAC coarse aggregate skeletons.
- SGC-based VCA measurement and dual volumetric correction enable target-driven PAC design.
- Skeleton proportion tailors PAC toward drainage/acoustic or heavy-load performance.

**Abstract:**

Porous asphalt concrete (PAC) relies on coarse- aggregate interlocking, but existing design methods lack quantitative skeleton evaluation and may introduce volumetric conversion errors. This study proposes a method combining California Bearing Ratio (CBR)-based mechanical skeleton optimization with coarse aggregate void-filling (CAVF) volumetric design, termed the CBR-CAVF method. An improved CBR test with continuous load displacement recording identified 40:60 and 55:45 blends of 10–15 mm and 5–10 mm aggregates as optimal, with *CBR*_5.0_ values of 35.9% and 39.8%. Voids in coarse aggregate were measured by Superpave gyratory compaction, and the coarse aggregate void-filling equation was corrected using a skeleton interference coefficient (α = 1.120) and effective binder volume to calculate gradations at a target air void content of 21.0%. Measured air voids differed from the target by no more than 0.4 percentage points. Rankings of Marshall stability, dynamic stability and the computed tomography (CT)-derived mean coordination number, skeleton ratio and aggregate contact ratio were consistent with the ranking of *CBR*_5.0_. Gradation 2 increased Marshall stability and dynamic stability by 43.8% and 18.3% over the empirical control, suiting heavy-load sections, while Gradation 1 achieved the highest permeability (7920 mL/min) and average normal-incidence sound absorption coefficient (0.356 over 500–1600 Hz), indicating potential benefits for drainage- and noise reduction-oriented applications.

## 1. Introduction

Porous asphalt concrete (PAC) is widely used to improve pavement drainage and wet weather safety, particularly where heavy rainfall increases the risk of surface ponding and hydroplaning [1]. PAC normally contains 18% to 25% design air voids and develops an interconnected pore network. This structure supports rapid surface drainage, improves skid resistance on wet pavement, reduces splash and spray and attenuates air pumping noise [2]. These benefits must be balanced against structural strength and durability [1,3]. With limited fine aggregate filling and mastic cohesion, PAC relies strongly on coarse aggregate contacts and interlock to transfer traffic loads. Insufficient skeleton stability can promote particle slippage and rutting, while the connected voids that facilitate drainage also expose the binder–aggregate interfaces to water. Raveling is a dominant early distress of porous asphalt pavement [4]. The combined effects of aging, moisture damage and traffic loading can cause progressive aggregate loss and shorten service life [5]. A central design challenge is therefore to maintain the open pore network required for drainage while providing a stable load-bearing skeleton and sufficient binder retention. This balance requires attention to both the mechanical contribution of the coarse aggregate assembly and the volumes of the constituents occupying its voids.

The mainstream PAC design methods currently used worldwide [6,7,8,9,10] include the open-graded friction course (OGFC) design method proposed by the National Center for Asphalt Technology (NCAT), the Bailey method proposed by the Illinois Department of Transportation, the recipe-type empirical design approach used in Europe under EN 13108-7:2016 [9] and the Marshall method widely adopted in China and Japan. These methods provide useful frameworks for PAC proportioning. The present study focuses on two aspects that warrant further attention: direct mechanical comparison of coarse aggregate blends before mixture preparation and volumetric calculation that accounts for the selected compaction state and effective binder volume.

For skeleton evaluation, *VCA*_mix_ denotes the voids in coarse aggregate within the compacted mixture, and *VCA*_DRC_ denotes the voids in the dry-rodded coarse aggregate skeleton. The criterion *VCA*_mix_ ≤ *VCA*_DRC_ [11] indicates stone-on-stone contact but does not quantify the relative bearing capacity of different coarse aggregate blends. Similarly, Marshall stability reflects the combined effects of skeleton interlock and mastic bonding rather than the contribution of the coarse aggregate alone. For volumetric design, proportioning based on dense filling must be adapted to the void-preserving objective of PAC. Mass-based gradation control also needs to account for differences in aggregate density. Although the Bailey method considers aggregate packing on a volumetric basis, PAC proportioning additionally requires the effective binder and target air voids to be incorporated into the volumetric balance. These considerations motivate a design procedure that connects mechanical skeleton selection with the calculation of constituent volumes.

Two lines of research have been pursued in response to these limitations. The first concerns characterization and evaluation of the skeleton structure. X-ray computed tomography (CT), digital image processing and discrete element simulation have been used to describe particle contacts, coordination numbers and contact networks [12]. Studies of gyratory compaction have linked more complete skeleton interlock to higher skeleton strength [13], while other studies have optimized gradations using inter-particle contact forces and unbalanced forces [14,15]. These approaches provide structural and mechanical insight, although they commonly involve characterization after mixture compaction or numerical simulation. A complementary laboratory screening step could provide direct comparisons of coarse aggregate bearing resistance before mixture preparation, with subsequent verification linking the selected skeletons to the structure and performance of the compacted mixtures.

The second line concerns volumetric gradation design. Fine-particle filling can contribute to a more compact internal structure in aggregate–binder composites [16]. In PAC proportioning, the volumes of fine aggregate and mastic must therefore be balanced with the air voids retained within the coarse aggregate skeleton. The coarse aggregate void-filling (CAVF) method has been applied to stone matrix asphalt (SMA), large-stone asphalt mixtures and porous asphalt mixtures [17]. It calculates constituent proportions from the target air void content and the voids available within the coarse aggregate skeleton. Its application to PAC requires attention to be paid to the difference between the dry-rodded skeleton state and the compacted mixture, the interference of fine aggregate and mastic with coarse aggregate packing and binder absorption. Accounting for these factors can improve the correspondence between the calculated composition and the volumetric state of the mixture. Combining this volumetric calculation with mechanical skeleton screening provides the basis for the procedure investigated here.

This study combines California Bearing Ratio (CBR)-based coarse aggregate screening with a modified CAVF volumetric calculation, referred to as the CBR-CAVF method. PAC-13 mixtures prepared with a fixed set of materials and a target air void content of 21.0% were selected as a case study to evaluate the feasibility of the proposed procedure under the investigated conditions. Coarse aggregate blends without asphalt binder were first compared using *CBR*_5.0_, the bearing ratio at a penetration depth of 5.0 mm. Superpave gyratory compaction (SGC) was then used to measure coarse aggregate skeleton voids and prepare mixture specimens. A skeleton interference coefficient and the effective binder volume were introduced into the volumetric calculation to determine constituent proportions. Experimental verification combined macroscopic pavement performance tests, CT analysis of the coarse aggregate contact structure and normal-incidence sound absorption measurements. Two CBR-selected gradations were compared with a conventional empirical control using the same material sources and asphalt–aggregate ratio, and accompanying differences in constituent proportions were considered when interpreting the measured performance. Its scientific value lies in experimentally examining the correspondence between coarse aggregate bearing resistance, contact structure in the compacted mixture and measured mechanical performance, alongside the agreement between target and measured air void contents. In practical terms, it provides a calculation route from a selected coarse aggregate blend and a prescribed air void content to constituent proportions, while the subsequent laboratory tests support comparison of candidate mixtures according to drainage, mechanical performance and sound absorption.

## 2. Materials and Methods

### 2.1. Raw Materials

In porous asphalt mixtures, the load is transferred between coarse aggregate particles through point contacts, so the binder must possess excellent adhesion and aging resistance in order to resist particle raveling under the coupled action of traffic loading and hydrodynamic pressure [18]. An SK high-viscosity modified asphalt supplied by Guangdong Huate Asphalt Co., Ltd. (Jiangmen, China) was used in this study. Its dynamic viscosity at 60 °C reaches 401,388 Pa·s, far higher than that of conventional modified asphalt, and it therefore provides sufficient bonding strength and raveling resistance for the large-void skeleton structure. Granular lignin fiber supplied by Shanghai Gongrui Industrial Co., Ltd. (Shanghai, China), with a mean length of 0.5 mm, was added to the mixture to further improve the reinforcing and thickening effects of the asphalt mastic and to suppress binder drain-down [19]. The properties of the asphalt were determined by the corresponding methods in JTG 3410-2025 [20] and those of the fiber by the corresponding methods in JT/T 533-2020 [21]. The main performance indices are given in Table 1.

**Figure 1:**
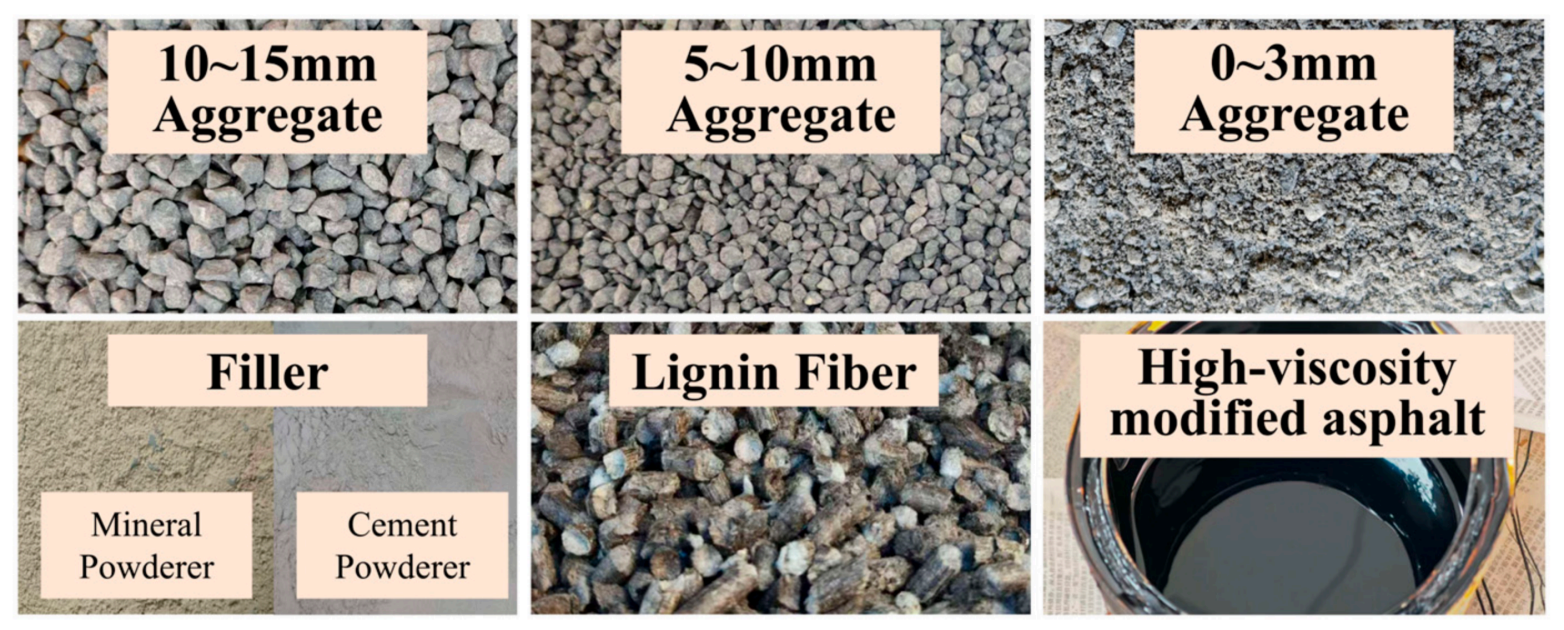
Constituent materials of the PAC mixture.

Coarse aggregate plays a decisive role in transferring load through interlock in the PAC skeleton. Diabase supplied by Youxin Quarry (Guigang, China), with good compressive and abrasion resistance was used as both coarse and fine aggregate. The aggregate crushing value and the Los Angeles abrasion loss of the coarse aggregate were 10.6% and 11.7%, the water absorption was 0.5% and the flat and elongated particle content was 4.6%, which ensured the mechanical stability of the skeleton under heavy loading. The fractions used were 10–15 mm and 5–10 mm. The fine aggregate had a size range of 0–3 mm and a sand equivalent of 72%. Dry and clean limestone mineral filler supplied by Heyuan Dashun Building Materials Co., Ltd. (Heyuan, China) was used. Part of the filler was replaced by P·O 42.5R Portland cement supplied by Guangdong GITIC Green Island Cement Company Limited (Yunfu, China) in order to strengthen the resistance of the mixture to moisture damage. The properties of the aggregates and the filler were determined by the corresponding methods in JTG 3432-2024 [22], and the physical properties of the mineral components are summarized in Table 2. The constituent materials used to prepare the PAC mixture are shown in Figure 1.

### 2.2. CBR-Based Evaluation of Coarse Aggregate Skeleton Interlocking

Load transfer in porous asphalt mixtures depends mainly on contact interlock and friction between coarse aggregate particles. If the coarse aggregate combination is too coarse, the contact points within the skeleton are too few; if it is too fine, small particles interfere with the effective contact between large particles. Either case weakens the skeleton interlock. It is therefore necessary to establish an evaluation method that directly reflects the load-bearing capacity of the coarse aggregate skeleton and that can be used to select the optimum skeleton proportion.

The California Bearing Ratio (CBR) test is an established method for evaluating the bearing capacity of soils and unbound granular materials. Its penetration resistance reflects the mechanical response of the particle assembly, including the effects of contact, friction and rearrangement. In this study, the coarse aggregate blends were tested without asphalt binder and therefore constituted unbound granular assemblies. The established CBR approach was therefore adapted to compare their load-bearing capacity and to guide coarse aggregate skeleton selection for the subsequent CAVF volumetric design. For coarse aggregate skeletons composed at different mass ratios, and under identical sample preparation, loading conditions and penetration depth, a higher CBR value indicates a stronger ability of the combination to resist particle slippage and rearrangement, providing a mechanical basis for comparing skeleton stability. The bearing ratio at 5.0 mm (*CBR*_5.0_) was selected as the screening index, using one of the conventional CBR reference penetrations as a common basis for comparison. Following the definition of the bearing ratio and the standard load intensity specified in T 0134-2019 of JTG 3430-2020 [23], the measured penetration pressure of the coarse aggregate skeleton was standardized, and the bearing ratio *CBR*_L_ of the coarse aggregate skeleton at different penetration depths was calculated by Equation (1):

(1) $$CBR_{L}=\frac{F_{L}\times 100\%}{A_{p}\times P_{L}}$$

where *CBR_L_* is the bearing ratio of the coarse aggregate skeleton at a penetration depth of *L* mm, %; *F*_L_ is the corresponding load measured on the coarse aggregate skeleton, N; *P_L_* is the corresponding standard load intensity, MPa, which equals 10.5 MPa at *L* = 5.0 mm; and *A_p_* is the cross-sectional area of the penetration piston, mm^2^.

The widely used PAC-13 porous asphalt mixture was taken as the example. In gradation design, the coarse aggregate skeleton is usually composed of the 10–15 mm and 5–10 mm fractions so as to reduce the interference of small particles with the skeleton. A preliminary screening of coarse aggregate combinations was first carried out at mass proportion intervals of 10%: the mass proportion of the 10–15 mm fraction was varied from 0% to 100% in steps of 10% (and the 5–10 mm fraction correspondingly from 100% to 0%), giving 11 combinations in total. Three replicate samples were prepared for each combination, each containing approximately 4 kg of coarse aggregate. Every sample was thoroughly mixed before the CBR test was carried out.

The samples were placed in a CBR mold with an inner diameter of 152 mm and a height of 166 mm. Each combination was charged into the mold in three layers of essentially equal height, and each layer was rodded uniformly 25 times with a steel tamping rod. This rodding provided preliminary compaction to facilitate placement of the surcharge plates and subsequent penetration loading. The same preparation procedure was applied to all blends to establish a common basis for comparing their penetration resistance under the prescribed test conditions. After the three layers had been placed, the sample surface was leveled, and eight surcharge plates, each with a mass of 625 g, were placed sequentially on top of the aggregate to simulate overburden confinement and reduce local disturbance during loading. The mold was then placed on the loading platform of an MTS universal testing machine (model MTS-810; MTS Systems Corporation, Eden Prairie, MN, USA). A penetration piston 50 mm in diameter was installed and the loading axis was lowered until it contacted the piston and produced a small initial load, at which point the reading was zeroed. During loading, the machine applied a continuous displacement-controlled rate of 1 mm/min until the penetration depth reached 5.0 mm, and the value measured at this depth, *CBR*_5.0_, was taken as the evaluation index.

**Figure 2:**
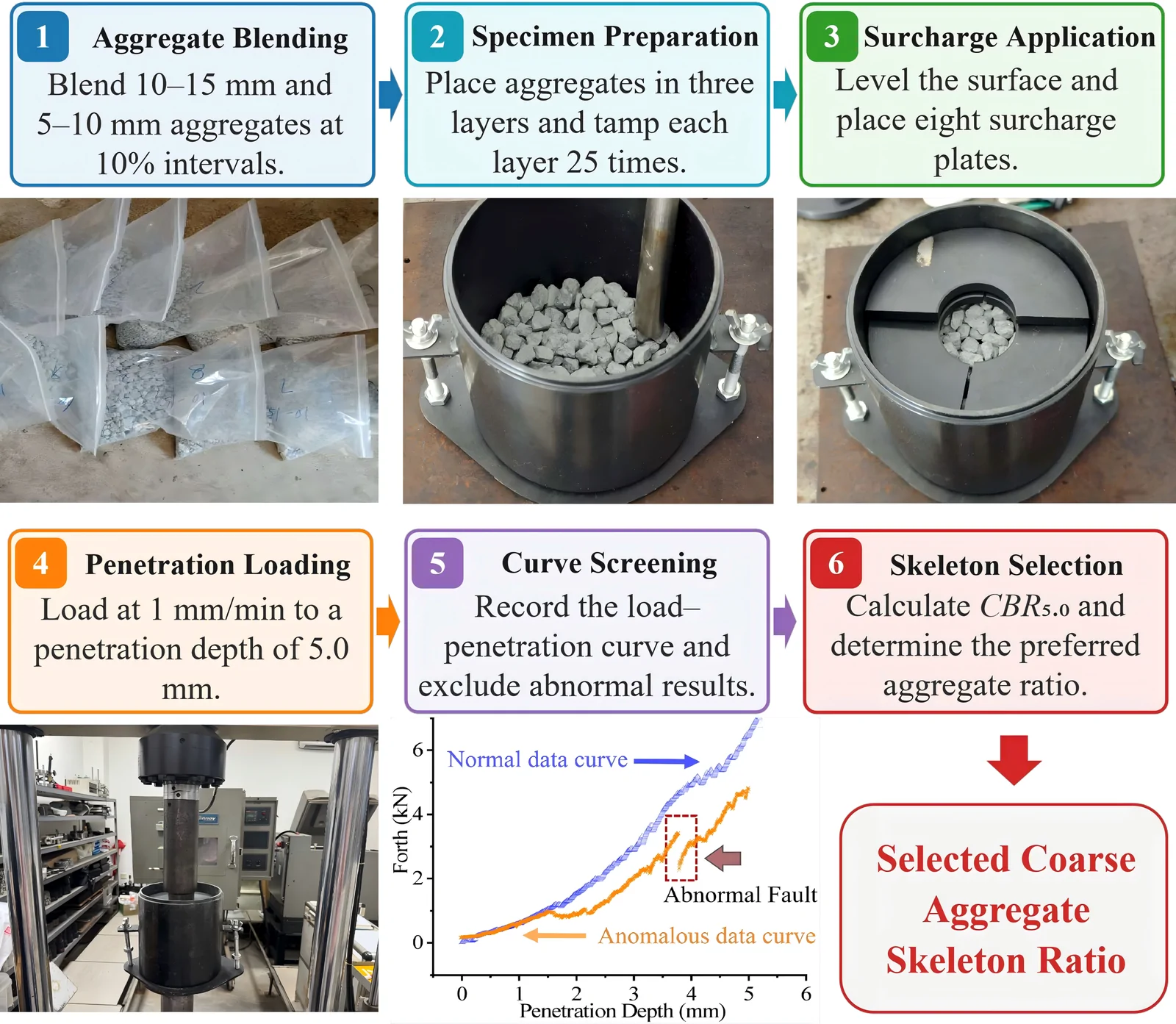
Test procedure for determining the coarse aggregate blending ratio based on the CBR test.

The relationship between penetration depth and load was recorded over the whole loading process by the MTS universal testing machine, whose load acquisition accuracy is 0.5% of full scale and displacement acquisition accuracy 0.01 mm. In this study, qualitative curve screening identified an abnormal response as a distinct, abrupt load drop interrupting an otherwise smoothly increasing load–penetration curve while penetration continued, followed by a gradual increase in load from the reduced level (Figure 2). This pattern was interpreted as possible evidence of pronounced particle rearrangement, local collapse or particle crushing inside the sample. The corresponding result was excluded from the skeleton screening, and the sample was discarded and remade for repeat testing. The reported *CBR*_5.0_ values therefore characterize the specimens retained under this screening procedure. The complete test procedure for determining the coarse aggregate blending ratio is summarized in Figure 2.

After the preliminary screening, and according to the *CBR*_5.0_ trend of the coarse aggregate skeleton with the proportion of the 10–15 mm fraction, a refined series of tests with a mass proportion interval of 5% was carried out in the neighborhood of the peak. The coarse aggregate blending ratios used in the subsequent CAVF volumetric design were then determined by combining the measured peak of *CBR*_5.0_, the trend of the locally fitted curve and the convenience of the values for engineering mix proportioning.

### 2.3. Specimen Compaction and Voids in Coarse Aggregate (VCA) Measurement by Superpave Gyratory Compaction (SGC)

The laboratory compaction method used for porous asphalt mixtures directly affects the spatial arrangement, contact state and void structure of the coarse aggregate. In the field, PAC is compacted mainly by the static kneading action of double-drum rollers, whereas the instantaneous impact of Marshall compaction readily causes crushing and local rearrangement of the coarse aggregate, so that the compacted state departs from the design intention. Superpave gyratory compaction (SGC) produces shearing and kneading effects through gyration of the mold at a small angle under constant vertical pressure. Its compaction mechanism is closer to field rolling and it causes less impact damage to the coarse aggregate, which helps to preserve the open-graded structure and the skeleton characteristics of PAC.

On this basis, SGC was adopted in the present study to prepare the PAC specimens using a G2 Superpave gyratory compactor (Pine Test Equipment, Inc., Grove City, PA, USA). Before mixing, the target preheating temperatures for the aggregates and high-viscosity modified asphalt were set at 190 °C and 180 °C, respectively. The cumulative mixing time after asphalt addition was 180 s, to ensure uniform distribution of the constituents and thorough coating of the aggregates. The mixture discharge temperature was controlled at 180 ± 5 °C [8]. The mixed material was charged into the gyratory compaction mold and compacted according to T 0736 in JTG 3410-2025 [20], with a mold inner diameter of 100 mm [24], a vertical pressure of 600 kPa, a gyration angle of 1.16°, a gyration speed of 30 r/min and 50 gyrations [8,25]. The specimens were used for the subsequent testing of void structure, drainage performance, mechanical properties, raveling resistance and moisture stability.

SGC was further used in this study to determine the voids in the coarse aggregate skeleton for volumetric design, replacing the conventional dry-rodded method. The conventional method compacts the coarse aggregate layer by layer with a manual tamping rod [26]. In the present procedure, manual rodding was used for preliminary compaction in the CBR screening, whereas SGC was used for skeleton void measurement and mixture specimen preparation. Compacting the coarse aggregate combination by SGC yields the voids in the coarse aggregate skeleton under a confinement state closer to that of specimen preparation [27], which improves the rationality of the skeleton void parameter used in the subsequent CAVF volumetric design. Each coarse aggregate combination selected by the CBR test was charged into a gyratory compaction mold and compacted using the same mold inner diameter, vertical pressure, gyration angle, gyration speed and number of gyrations as used for the PAC specimens. The height and mass of the compacted skeleton were measured, and, with reference to the void calculation relationship in ASTM C29/C29M-23 [28], the voids in coarse aggregate *VCA*_SGC_ were calculated by Equations (2) and (3) as an important input parameter for the subsequent volumetric design:

(2) $$VCA_{\mathrm{SGC}}=(1-\frac{\rho_{\mathrm{SGC}}}{\gamma_{\mathrm{cab}}\rho_{w}})\times 100\%$$

(3) $$\rho_{\mathrm{SGC}}=\frac{m_{\mathrm{ca}}}{A_{m}h_{\mathrm{ca}}}$$

where *VCA*_SGC_ is the measured void content in the coarse aggregate skeleton after 50 gyrations, %; *γ*_cab_ is the combined bulk specific gravity of the coarse aggregate blend on a dry basis; *ρ*_SGC_ is the bulk density of the coarse aggregate skeleton after gyratory compaction, g/cm^3^; *ρ*_w_ is the density of water, g/cm^3^; *m*_ca_ is the mass of the coarse aggregate, g; *A*_m_ is the internal cross-sectional area of the compaction mold, cm^2^; and *h*_ca_ is the mean height of the coarse aggregate, cm.

### 2.4. Gradation Design Using the Modified CAVF Method

#### 2.4.1. Principle of the CAVF Method

The coarse aggregate void-filling (CAVF) method was proposed by Zhang et al. [29,30]. The coarse aggregate forms the load-bearing skeleton, while the other constituents fill its voids and leave the target air voids. The fine aggregate proportion is back-calculated from the volumetric balance once the skeleton and other constituent volumes are specified.

The mineral aggregate mass balance is *q*_c_ + *q*_f_ + *q*_p_ = 100, where *q*_c_, *q*_f_ and *q*_p_ are the mass percentages of coarse aggregate, fine aggregate and filler in the total mineral aggregate, respectively. The corresponding volumetric relationship is expressed by Equation (4):

(4) $$\frac{q_{f}}{\gamma_{f}}+\frac{q_{p}}{\gamma_{p}}=\frac{q_{c}}{100\rho_{\mathrm{DRC}}}(VCA_{\mathrm{DRC}}-V_{V}-V_{b})$$

where *γ*_f_ and *γ*_p_ are the apparent relative densities of fine aggregate and filler, respectively; *ρ*_DRC_ is the dry-rodded bulk density of the coarse aggregate, g/cm^3^; *VCA*_DRC_ is the voids in the coarse aggregate skeleton in the dry-rodded condition, %; *V*_v_ is the design air void content of the mixture, %; and *V*_b_ is the volume of asphalt, %.

#### 2.4.2. Modifications: Skeleton Interference Coefficient and Effective Binder Volume

During mixing and compaction, fine aggregate, mineral filler, fiber and asphalt mastic can alter coarse aggregate packing. The resulting skeleton voids within the mixture may therefore exceed the *VCA*_SGC_ measured on coarse aggregate compacted alone.

**Figure 3:**
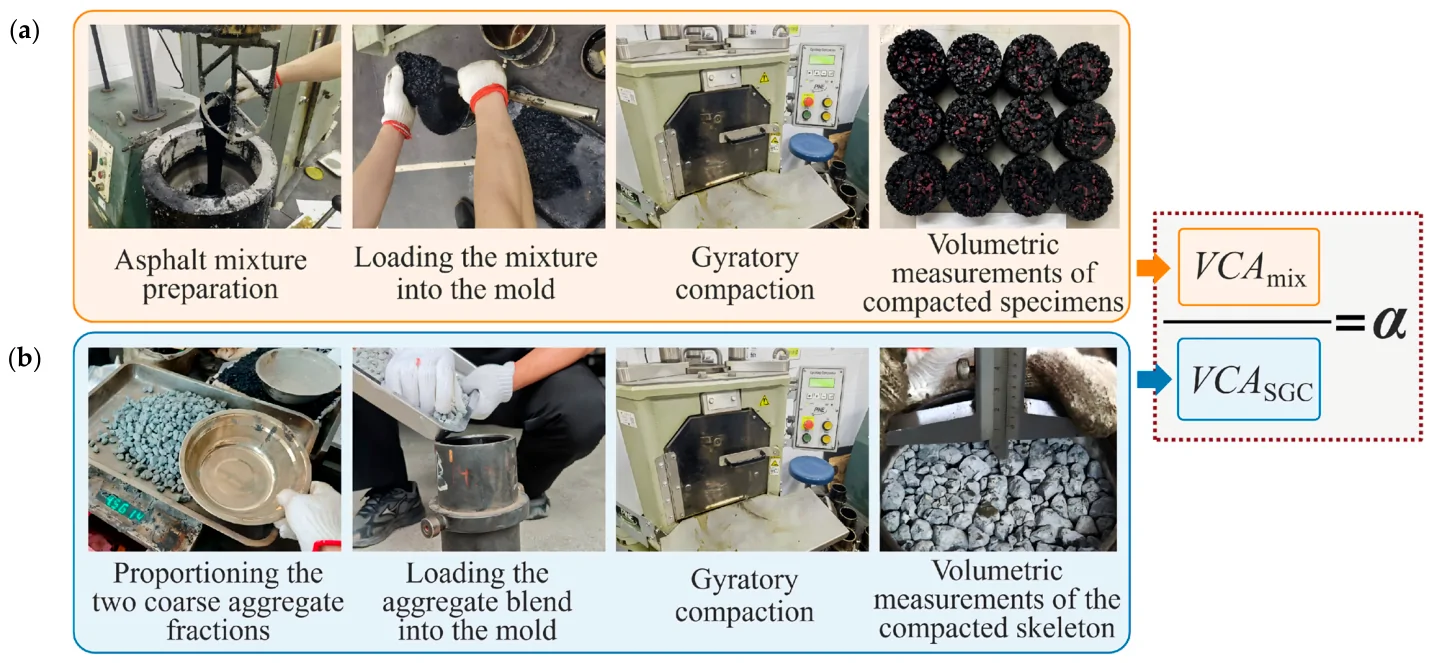
Experimental procedure for calibrating the skeleton interference coefficient α: (**a**) preparation and volumetric measurements of the reference mixture (Gradation 3); (**b**) gyratory compaction and volumetric measurements of the corresponding coarse aggregate blend.

To correct this difference, a skeleton interference coefficient α was introduced to account for the error caused by the interference and to convert the *VCA*_SGC_ measured under gyratory compaction into the actual voids in the coarse aggregate skeleton within the mixture, *VCA*_mix_. In this study, α is defined as the dimensionless ratio *VCA*_mix_/*VCA*_SGC_ for a reference mixture and its corresponding coarse aggregate blend. Its value is determined from volumetric measurements of the two packing states under the adopted SGC conditions, providing an empirical correction based on the materials and compaction conditions used. Figure 3 illustrates the experimental procedure for this calibration using Gradation 3 and its corresponding coarse aggregate blend under the same SGC settings. The calibration results are reported in Section 3.2.

Because asphalt absorbed by the aggregate cannot fully participate in particle bonding and asphalt film formation, the effective binder volume *V*_be_ was used in the calculation of skeleton void filling. *V*_be_ was calculated from the target asphalt film thickness and the absorption characteristics of the aggregate according to Equation (B.6.8-3) of JTG F40-2004 [31]. With *VCA*_mix_ and *V*_be_ introduced, the CAVF design relationship is further modified into Equation (5):

(5) $$\frac{q_{f}}{\gamma_{f}}+\frac{q_{p}}{\gamma_{p}}=\frac{q_{c}}{100\rho_{\mathrm{SGC}}}(\alpha VCA_{\mathrm{SGC}}-V_{V}-V_{\mathrm{be}})$$

#### 2.4.3. CBR-CAVF Design Procedure

**Figure 4:**
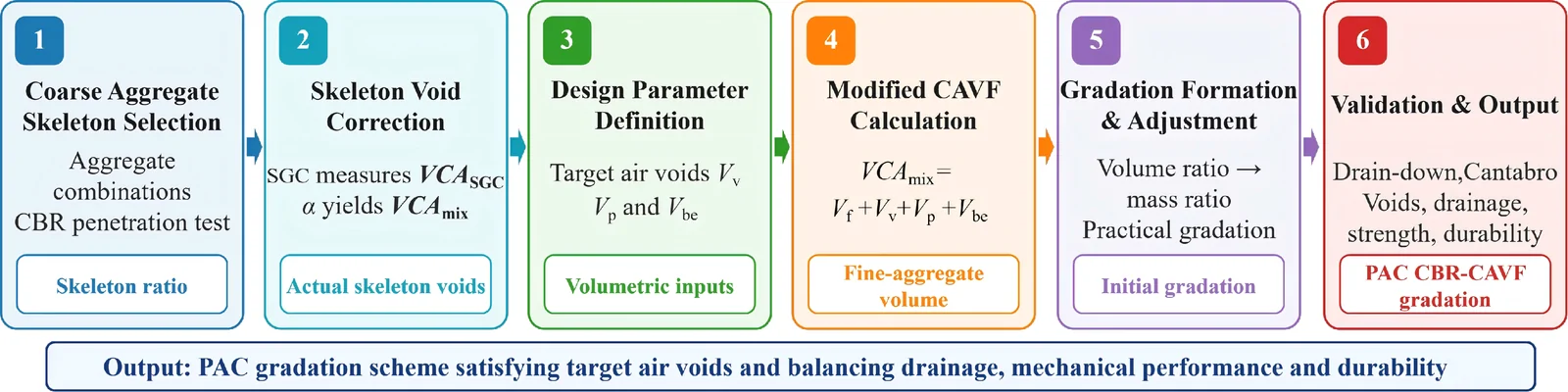
Flowchart of the design method for PAC mixture based on the CBR-CAVF approach.

On the basis of the above concept, the CBR-CAVF gradation design procedure established in this study is shown in Figure 4. The coarse aggregate skeleton proportion is first selected by the CBR test. *VCA*_SGC_ is then measured by gyratory compaction and corrected by the skeleton interference coefficient to give *VCA*_mix_. The target air void content *V*_v_ is subsequently set according to the functional requirements, and the volume of filler and fiber *V*_p_ and the effective binder volume *V*_be_ are determined. The volume of fine aggregate *V*_f_ is back-calculated from the volumetric balance and converted into mass proportions of the mineral aggregate. After rounding for engineering practice, the asphalt content is verified by the binder drain-down and Cantabro tests, which completes the gradation design and the performance verification. The detailed procedure is presented in Section 3.

### 2.5. Performance Tests and Structural Characterization

In order to verify the effectiveness of the CBR-CAVF design method systematically, three aspects of the mixtures were tested: macroscopic pavement performance, meso-scale skeleton structure and acoustic function. Except for the wheel-tracking test, in which 300 mm × 300 mm × 50 mm slab specimens formed by a roller compactor were used, all specimens were φ100 mm × 63.5 ± 1.3 mm cylinders prepared by SGC. Unless otherwise stated, numerical test results are reported as the mean ± standard deviation (SD), with the number of replicate results specified for each test. Figures were prepared using Origin 2021 (OriginLab Corporation, Northampton, MA, USA) and WPS Presentation (version 12.1.0; Zhuhai Kingsoft Office Software Co., Ltd., Zhuhai, China).

#### 2.5.1. Macroscopic Pavement Performance Tests

The macroscopic pavement performance tests covered six categories of indices: void structure, drainage capacity, mechanical strength, high-temperature stability, raveling resistance and moisture stability. Unless otherwise stated, they were carried out by the corresponding methods given in JTG 3410-2025 [20], ‘Standard Test Methods of Asphalt and Asphalt Mixture for Highway Engineering’. The connected air void content was determined according to Equations (6) and (7) [32]:

(6) $$V_{c}=\frac{V_{m}-V_{t}}{V_{m}}\times 100\%$$

(7) $$V_{t}=\frac{m_{d}-m_{w}}{\rho_{w}}$$

where *V*_c_ is the connected air void content, %; *V*_m_ is the volume of the specimen, cm^3^; *V*_t_ is the combined volume of the mixture and the closed voids, cm^3^; *m*_d_ is the mass of the specimen in the dry state at room temperature, g; *m*_w_ is the mass of the specimen in water, g; and *ρ*_w_ is the density of water, g/cm^3^.

#### 2.5.2. CT-Based Meso-Structural Analysis of the Aggregate Skeleton

In order to explain the differences in macroscopic mechanical performance and durability between the mixtures of different gradations at the meso scale, the internal structure of the specimens of the three gradations was characterized by CT scanning. The equipment comprised a Compact-225 system (YXLON International GmbH, Hamburg, Germany) with a maximum imaging resolution of 1024 × 1024 pixels and an interlayer scanning resolution of 0.02 mm. One representative specimen of each gradation was independently scanned (three specimens in total). For each specimen, 12 cross-sections were selected at intervals of 5 mm over the depth range of 5–60 mm along the specimen height for coarse aggregate identification, contact determination and statistical analysis of the skeleton network. Thus, 12 cross-sections per gradation and 36 cross-sections in total were analyzed.

**Figure 5:**
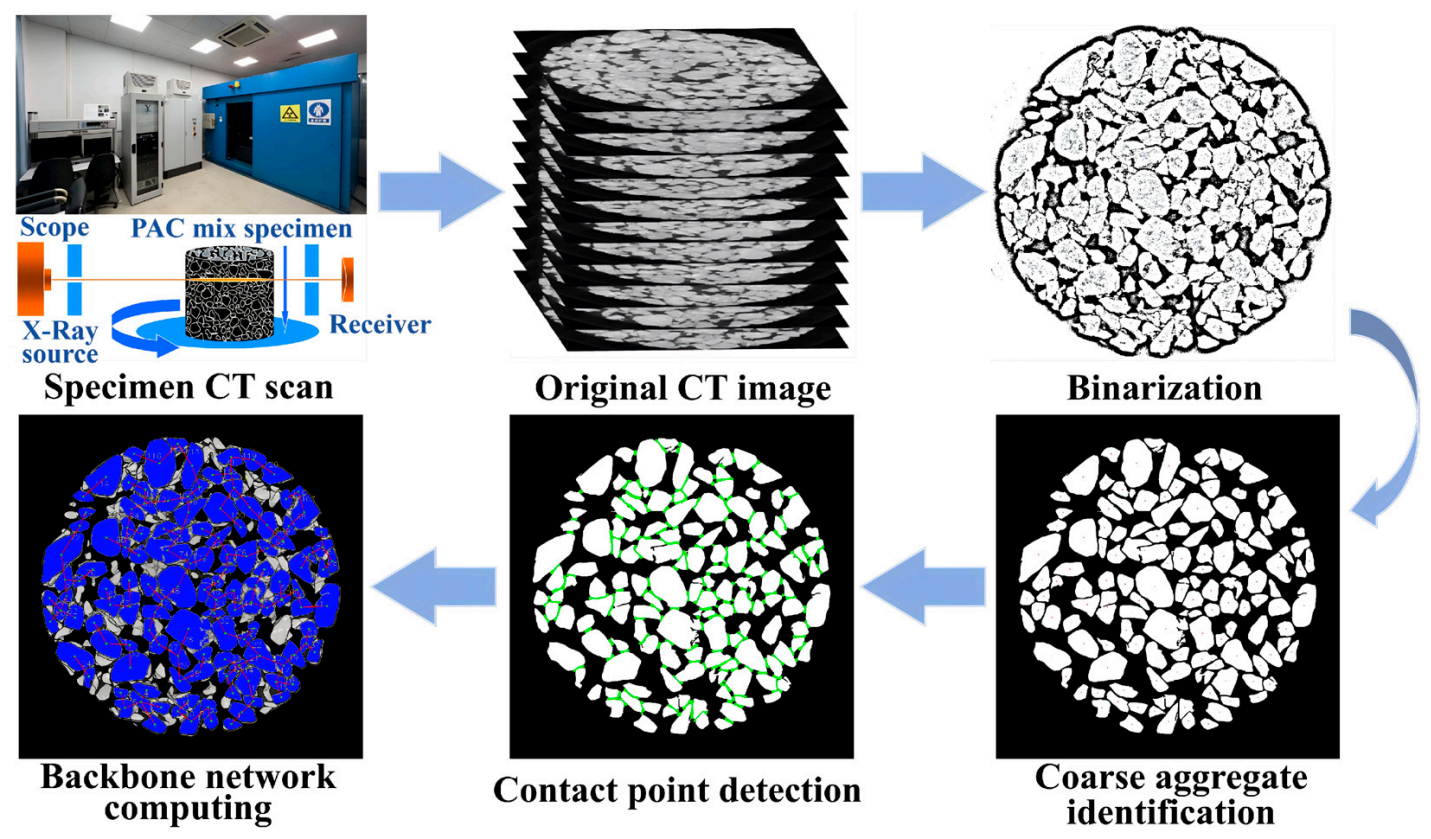
CT image analysis procedure.

The aggregate skeleton was analyzed using in-house software developed at South China University of Technology (Guangzhou, China) and implemented in MATLAB R2021a (The MathWorks, Inc., Natick, MA, USA). The CT image analysis procedure is shown in Figure 5. The PAC specimens were first scanned layer by layer. The original CT cross-sectional images were then subjected to gray-level correction, binarization and noise suppression, and the coarse aggregate regions were identified by threshold segmentation and morphological processing. The contact relationships and the effective skeleton region were subsequently determined from the minimum distance between the boundaries of adjacent particles. According to previous studies [33,34], a contact threshold equal to 23% of the minimum calculated particle size is reasonable, that is, 0.54 mm. When the boundary spacing between two adjacent coarse aggregate particles is not greater than this value, an effective contact point is identified.

In order to evaluate the interlocking state of the coarse aggregate skeletons of the different gradations quantitatively, the number of contact points *Q*_c_, the mean coordination number ${\bar{n}}_{c}$, the skeleton ratio *S* and the aggregate contact ratio *C* were used as indices of the coarse aggregate contact network. *Q*_c_ is the total number of contact points formed between coarse aggregate particles within a cross-section and reflects the overall number of contacts. ${\bar{n}}_{c}$, calculated as ${\bar{n}}_{c}=\frac{1}{N}\sum_{i=1}^{N}n_{i}$, is the average number of contacts per coarse aggregate particle and evaluates how fully the particles are in contact with one another. Here, *N* is the number of coarse aggregate particles within the cross-section, and *n_i_* is the number of contacts of the *i*-th coarse aggregate particle. The skeleton ratio *S*, calculated as *S* = *A*_s_ / *A*_t_ × 100%, characterizes the proportion of the cross-sectional area of the specimen occupied by coarse aggregate that participates in the effective skeleton. The aggregate contact ratio *C*, calculated as *C* = *A*_s_ / *A*_c_ × 100%, characterizes the proportion of the coarse aggregate that participates in the effective skeleton contact network. Here, *A*_t_ is the total area of the specimen cross-section, *A*_s_ is the area of the coarse aggregate participating in the effective skeleton, and *A*_c_ is the total area of coarse aggregate within the cross-section; all three areas are expressed in mm^2^.

#### 2.5.3. Normal-Incidence Sound Absorption Measurement Using a Standing-Wave Tube

Normal-incidence sound absorption was measured to compare the acoustic response of the three PAC-13 gradations. Sound absorption arises from viscous and thermal energy losses in the pore network [35], and its frequency response depends on porosity and specimen thickness [36].

An AWA6290 standing-wave tube measurement system (Hangzhou Aihua Instruments Co., Ltd., Hangzhou, China) was used, and the tests were carried out in accordance with ISO 10534-1:1996 [37], covering a number of frequencies in the range of 100–1600 Hz. This method was selected because it allows frequency-dependent sound absorption to be compared between laboratory-prepared mixtures under controlled normal-incidence conditions.

Dense-graded asphalt concrete (AC-13) and SMA-13 were included as reference mixtures, using the median gradations of JTG F40-2004 [31] and asphalt–aggregate ratios of 4.8% and 6.0%, respectively. Two specimens were prepared for each of the five gradations by SGC with 50 gyrations. The specimens were 100 mm in diameter and their thickness was controlled at 63.5 ± 1.3 mm.

**Figure 6:**
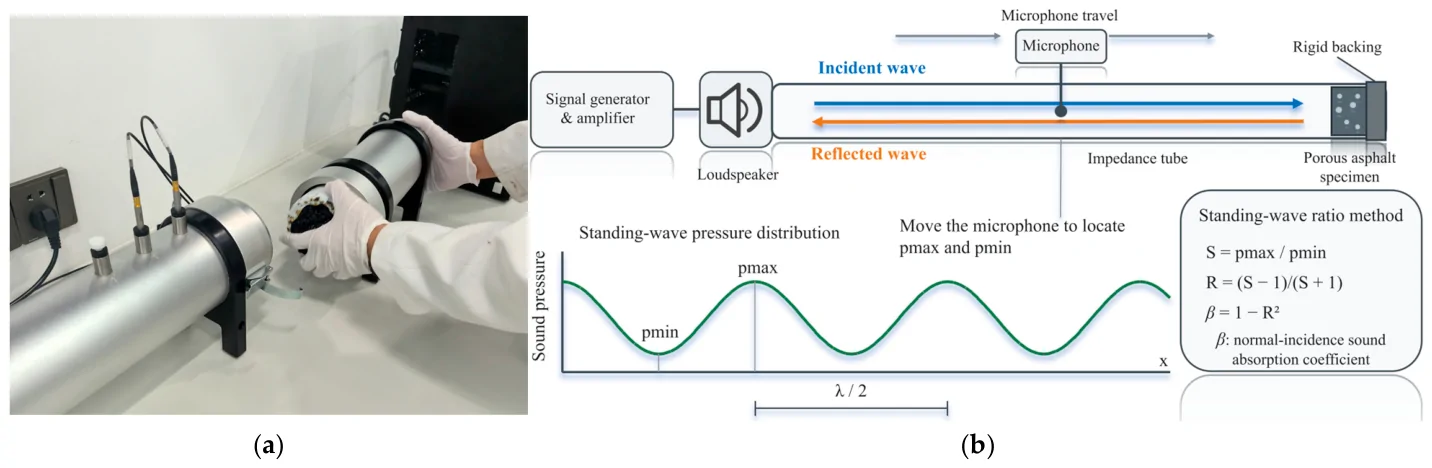
The normal-incidence sound absorption measurement: (**a**) specimen mounted in the standing-wave tube; (**b**) principle of measurement. In panel (**b**), S denotes the standing-wave ratio, R the magnitude of the pressure reflection coefficient, pmax and pmin the maximum and minimum sound pressure amplitudes, λ the wavelength and x the axial position along the tube.

Before testing, each specimen was sealed laterally to prevent leakage between the specimen and the tube wall and mounted against a rigid backing with no rear air cavity. Each specimen was tested at circumferential orientations of 0°, 120° and 240° to reduce the influence of surface heterogeneity and orientation. At each frequency, the six measurements from the two specimens were averaged to obtain the result for each gradation. The measured coefficients characterize absorption for the tested specimen geometry, frequency range and mounting conditions and are used here to assess relative acoustic potential, rather than to quantify field noise reduction. The test equipment and the measuring principle are shown in Figure 6a and Figure 6b, respectively.

During the test, the sound source emits a pure-tone signal that forms a standing-wave field inside the tube. Following ISO 10534-1:1996 [37], the normal-incidence sound absorption coefficient *β* (ranging from 0 to 1) at each frequency is calculated from the measured standing-wave ratio. The larger this value, the higher the proportion of the incident sound energy absorbed by the specimen.

Regarding the evaluation indices, the sound absorption coefficient–frequency curve was used to describe the frequency response of the mixtures of the three gradations, and the mean sound absorption coefficient over the test band $\bar{\beta }$, the peak sound absorption coefficient *β*_max_ and the frequency corresponding to the peak *f*_p_ were used for quantitative comparison. $\bar{\beta }$ is calculated by Equations (8) and (9); *β*_max_ is the maximum sound absorption coefficient among all the test frequencies; and *f*_p_ is the frequency corresponding to *β*_max_. At the *i*-th test frequency, the standing-wave ratio is calculated as *S_i_* = *p*_max,_*_i_* / *p*_min,_*_i_*, where *p*_max,_*_i_* and *p*_min,_*_i_* are the maximum and minimum sound pressures, respectively (Pa).

(8) $$\beta_{i}=1-{\left(\frac{S_{i}-1}{S_{i}+1}\right)}^{2}$$

(9) $$\bar{\beta }=\frac{1}{n}\sum_{i=1}^{n}\beta_{i}$$

where *β*_i_ is the normal-incidence sound absorption coefficient at the *i*-th test frequency; $\bar{\beta }$ is the mean sound absorption coefficient over the test band; and *n* is the number of test frequencies within the 500–1600 Hz band, which is the band in which the tire–pavement noise energy is most concentrated.

## 3. PAC-13 Mix Design Using the CBR-CAVF Method

### 3.1. Optimization of the Coarse Aggregate Blending Ratio

**Figure 7:**
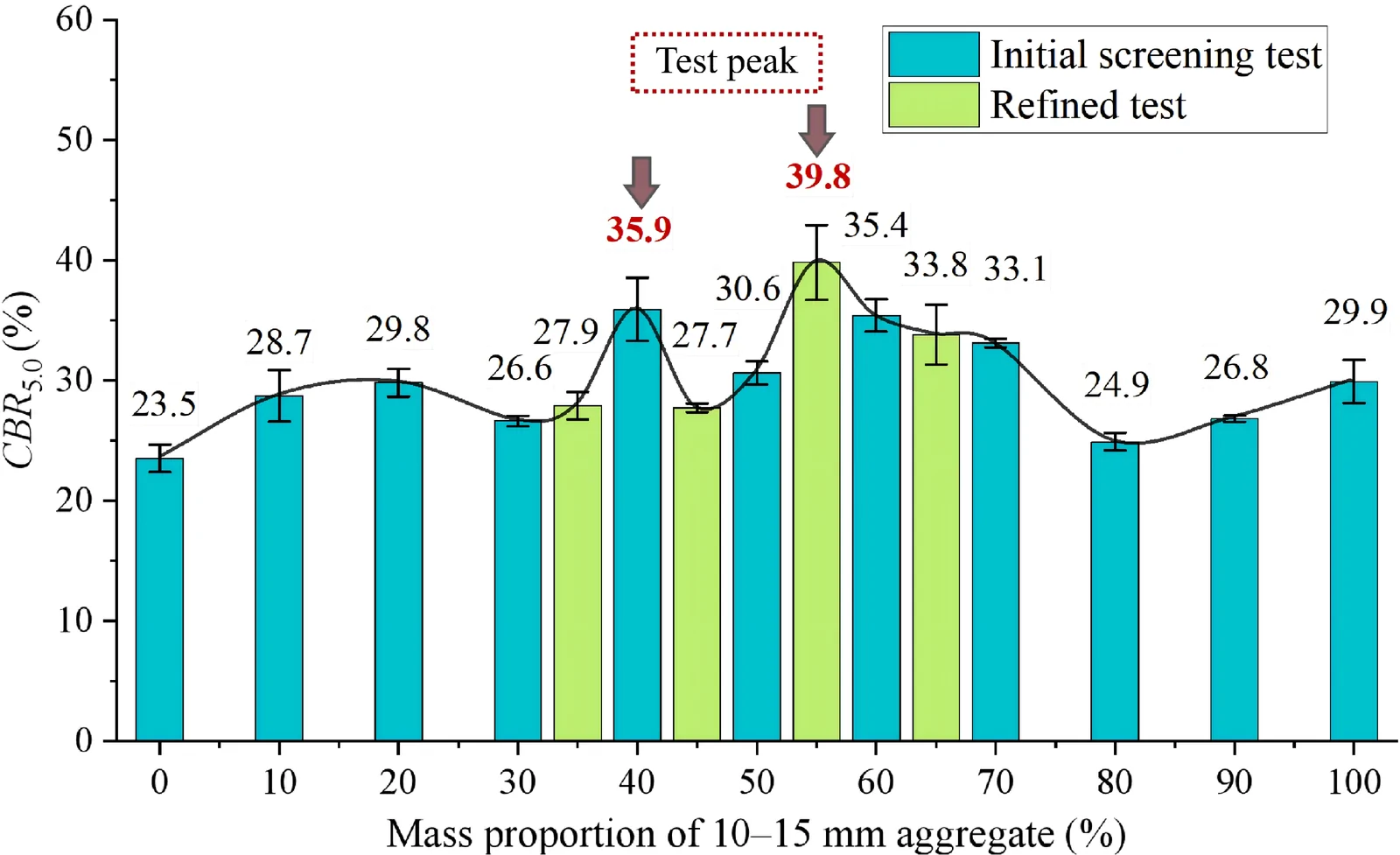
*CBR*_5.0_ test results of the coarse aggregate at different mass blending ratios. Bars show the mean *CBR*_5.0_ values, and error bars represent ± one standard deviation from three replicate samples for each combination (*n* = 3).

Following the method described in Section 2.2, preliminary screening tests were carried out on the 11 combinations of the 10–15 mm and 5–10 mm coarse aggregate fractions, and refined tests at intervals of 5% were then conducted on both sides of the peaks. Three replicate samples were prepared for each combination, and the mean value of *CBR*_5.0_ was taken as the index of skeleton interlocking strength. The test results are shown in Figure 7.

The preliminary screening results show that *CBR*_5.0_ fluctuates markedly as the mass proportion of the 10–15 mm fraction increases rather than varying monotonically with the proportion of either fraction. This behavior is consistent with the mutual interference mechanism of particle packing. When the proportion of large particles is low, the few large particles present actually disturb the dense packing of the surrounding small particles through the wall effect; when the proportion of large particles is too high, the small particles are too few to wedge into the gaps between the large particles and provide effective support, and the loosening effect reduces the number of effective contact points within the skeleton [38]. Only when the proportions of the two fractions are well matched, so that the secondary particles fit precisely into the voids of the primary skeleton and participate in load transfer, does the resistance of the skeleton to penetration deformation reach a local optimum [13].

The screening results reveal a first high-value region at a 10–15 mm proportion of 40% and a second one in the vicinity of 60%. Refined tests were therefore carried out around these two regions in order to identify representative proportions within the high-value ranges. The results show that the *CBR*_5.0_ of the coarse aggregate skeleton is relatively high at 10–15 mm proportions of 40% and 55%, reaching 35.9% and 39.8%, respectively, which is about 18.5% and 31.4% higher than the mean level of all the combinations tested. For these two selected combinations, the standard deviations were 2.6 and 3.1 percentage points (*n* = 3), corresponding to coefficients of variation of 7.2% and 7.8%, respectively.

Taking the screening results, the refined test results and the convenience of the values for engineering mix proportioning together, two coarse aggregate combinations with 10–15 mm and 5–10 mm at mass ratios of 40:60 and 55:45 were selected as the primary skeleton proportions for the subsequent CAVF volumetric design. The mixtures designed on the basis of these two coarse aggregate skeletons are hereafter denoted as Gradation 1 and Gradation 2, and a conventional empirical gradation was included as the control.

### 3.2. Determination of VCA and the Skeleton Interference Coefficient

Five replicate gyratory compaction tests were conducted for each coarse aggregate combination selected in Section 3.1. The mean *VCA*_SGC_ values were 36.23% and 37.11% for the 40:60 and 55:45 combinations, respectively, with coefficients of variation below 0.3%, indicating good repeatability under the adopted conditions. Moreover, the *VCA*_SGC_ of the 55:45 combination was 0.88 percentage points higher than that of the 40:60 combination, which reflects the larger void scale of a skeleton dominated by large particles and the consequent need to reserve a greater filling volume in the volumetric design. The individual and mean results are listed in Table 3.

In an actual mixture, the fine aggregate, mineral filler, fiber and asphalt mastic interfere with the arrangement of the coarse aggregate skeleton, so that the actual voids in the coarse aggregate skeleton within the mixture, *VCA*_mix_, differ from the *VCA*_SGC_ measured on the coarse aggregate alone under gyratory compaction. A skeleton interference coefficient *α* was therefore introduced to correct the skeleton voids quantitatively. In order to calibrate *α*, a porous asphalt mixture with the conventional empirical gradation (Gradation 3 in Section 3.4) was prepared using the same raw materials and the same compaction method, and *VCA*_mix_ was calculated by Equation (10) following the method for calculating the voids in the coarse aggregate skeleton of a specimen given in ASTM D7064/D7064M-21 [39].

(10) $$VCA_{\mathrm{mix}}=100-\frac{\gamma_{\mathrm{mb}}P_{\mathrm{ca}}}{\gamma_{\mathrm{cab}}}$$

where *γ*_mb_ is the bulk specific gravity of the mixture; *γ*_cab_ is the combined bulk specific gravity of the coarse aggregate fraction; and *P*_ca_ is the mass percentage of coarse aggregate in the total mass of the asphalt mixture, %.

The results show that the *VCA*_mix_ of the Gradation 3 mixture was 41.06 ± 0.13% (*n* = 5), while the *VCA*_SGC_ of the corresponding coarse aggregate combination measured by gyratory compaction was 36.65 ± 0.09% (*n* = 5). The skeleton interference coefficient obtained was *α* = *VCA*_mix_ / *VCA*_SGC_ = 1.120 ± 0.004 (*n* = 5).

The α calibrated from Gradation 3 was applied to Gradations 1 and 2 as an empirical approximation within the same material system, target air void range and gyratory compaction method. This was further supported by their identical asphalt–aggregate ratio, cement content and fiber dosage and similar 0.075 mm passing percentages (4.1–4.3%; Section 3.4, Table 6). However, the fine aggregate and mineral filler contents differed, so the use of a common α does not imply that the interference coefficient is independent of composition. The estimated *VCA*_mix_ values of 40.58% and 41.57% for the 40:60 and 55:45 combinations, respectively, were used in the subsequent volumetric calculations. The measured air void deviations of 0.16 and 0.36 percentage points for Gradations 1 and 2 (Section 4.1.1) provide support for the corrected volumetric design incorporating this approximation under the investigated conditions.

### 3.3. Volumetric Composition Calculation

The constituent volumes of the two CBR-CAVF mixtures were calculated using the modified volumetric relationship in Section 2.4 and the determined *VCA*_mix_ values.

(1) Target air void content. The target air void content was selected to balance drainage and durability, as excessive voids increase the risk of raveling and moisture damage [40]. JTG/T 3350-03-2020 [8] specifies a minimum design air void content of 18%, while values of at least 20% are commonly used in practice. Considering this requirement and the bonding capacity of the high-viscosity modified asphalt, *V*v = 21.0% was adopted for the present PAC-13 mixtures.
(2) Volume of filler and fiber. The filler and fiber contents were selected with reference to engineering experience with PAC-13 mixtures in South China. Gradation 1 contained 2.0% mineral filler, 1.0% cement and 0.3% lignin fiber. For Gradation 2, which had a higher *VCA*_mix_ and required more fine aggregate filling, 1.5% mineral filler was adopted as a design input to avoid an excessive powder volume; the cement and fiber contents were unchanged. The resulting 0.075 mm passing percentage differed from those of the other two gradations by no more than 0.2 percentage points (Section 3.4). Conversion using the constituent densities yielded *V*_p,1_ = 2.75% and *V*_p,2_ = 2.37%.
(3) Effective binder volume. The relevant Chinese specifications recommend an asphalt film thickness of no less than 14 μm [8], and recent research likewise indicates that the optimum film thickness balancing service performance against aging resistance is approximately 13–15 μm [41]. A target asphalt film thickness of 14 μm was therefore adopted in this study, and *V*_be_ = 9.25% was obtained by calculation according to Equation (B.6.8-3) of JTG F40-2004 [31] in combination with the specific surface area of the mineral aggregate and the absorption characteristics of the aggregate.

The fine aggregate volumes were then obtained as *V*_f_ = *VCA*_mix_ − *V*_v_ − *V*_p_ − *V*_be_, giving *V*_f,1_ = 7.58% and *V*_f,2_ = 8.95%.

On the basis of these calculations, the volumetric compositions of the two CBR-CAVF design gradations are summarized in Table 4.

### 3.4. Design and Control Gradations

Based on the volumetric proportions calculated in Section 3.3, and using the apparent relative densities of the individual aggregate fractions, the mineral filler and the cement, the two CBR-CAVF design schemes were converted from volumetric proportions into mass proportions of the mineral aggregate. The lignin fiber and the asphalt were both dosed as external additions and do not form part of the 100% mass composition of the mineral aggregate. Taking into account the weighing accuracy in actual mixing production and the convenience of engineering application, the mass proportions obtained from the preliminary calculation were rounded appropriately, without altering the principal skeleton proportions or the overall gradation characteristics, to give the two CBR-CAVF design gradations. In order to evaluate the effectiveness of the CBR-CAVF design method, a typical conventional gradation used on expressways in South China was included as the control and is denoted as Gradation 3. It employs the same raw materials and the same filler system. The mass compositions of the mineral aggregate for the three gradations are given in Table 5.

From the sieve analysis of each aggregate fraction, the combined percentages passing were calculated for the three gradations. The results are given in Table 6. All three gradations belong to the PAC-13 open-graded structure, and the differences between them are concentrated mainly in the coarse aggregate range of 4.75–13.2 mm. The percentage passing the 9.5 mm sieve decreases in the order Gradation 1 (65.2%) > Gradation 3 (60.5%) > Gradation 2 (55.1%), whereas the percentages passing the 2.36 mm and finer sieves are similar for the three gradations. The mineral filler and fine aggregate contents also differ: Gradation 2 contains 1.5% mineral filler and 13.0% fine aggregate, compared with 2.0% and 11.0%, respectively, in Gradations 1 and 3 (Table 5). These compositional differences are considered alongside the coarse aggregate skeleton when interpreting the mixture performance. In addition, the relative mass ratio of the 10–15 mm and 5–10 mm fractions in Gradation 3 is approximately 46.5:53.5, which is close to the 45:55 combination that gave a low *CBR*_5.0_ in the refined tests of Section 3.1. The coarse aggregate blend corresponding to Gradation 3 was also tested using the CBR procedure described in Section 2.2. Three replicate samples gave a mean CBR5.0 of 28.0% with a standard deviation of 2.2 percentage points. This feature makes Gradation 3 not only a control at the design method level, but also a mechanical reference for explaining the differences in pavement performance between the gradations from the standpoint of skeleton load-bearing capacity.

In summary, two PAC-13 gradations designed by the CBR-CAVF method (Gradation 1 and Gradation 2) and one conventional empirical control gradation (Gradation 3) were established. In the following sections, the drainage performance, mechanical properties, durability and meso-structural characteristics of the three mixtures are compared systematically under the same method of determining the asphalt content, the same specimen compaction conditions and the same performance testing system.

### 3.5. Determination of the Optimum Binder Content

The asphalt content of a porous asphalt mixture has to satisfy both raveling resistance and drain-down resistance requirements. When the asphalt–aggregate ratio is too low, the asphalt film on the aggregate surface is too thin, the bonding capacity between particles is reduced and the mixture is prone to raveling; when the asphalt–aggregate ratio is too high, the excess binder tends to drain down and may impair the connected void structure. Internationally, the Cantabro abrasion test and the binder drain-down test are used together to determine a reasonable range of asphalt content for PAC [39,42,43]. After the gradations had been determined, the Schellenberg binder drain-down test and the Cantabro abrasion test were therefore carried out according to T 0732 and T 0733 of JTG 3410-2025 [20] in order to verify the asphalt content of the three gradations.

**Figure 8:**
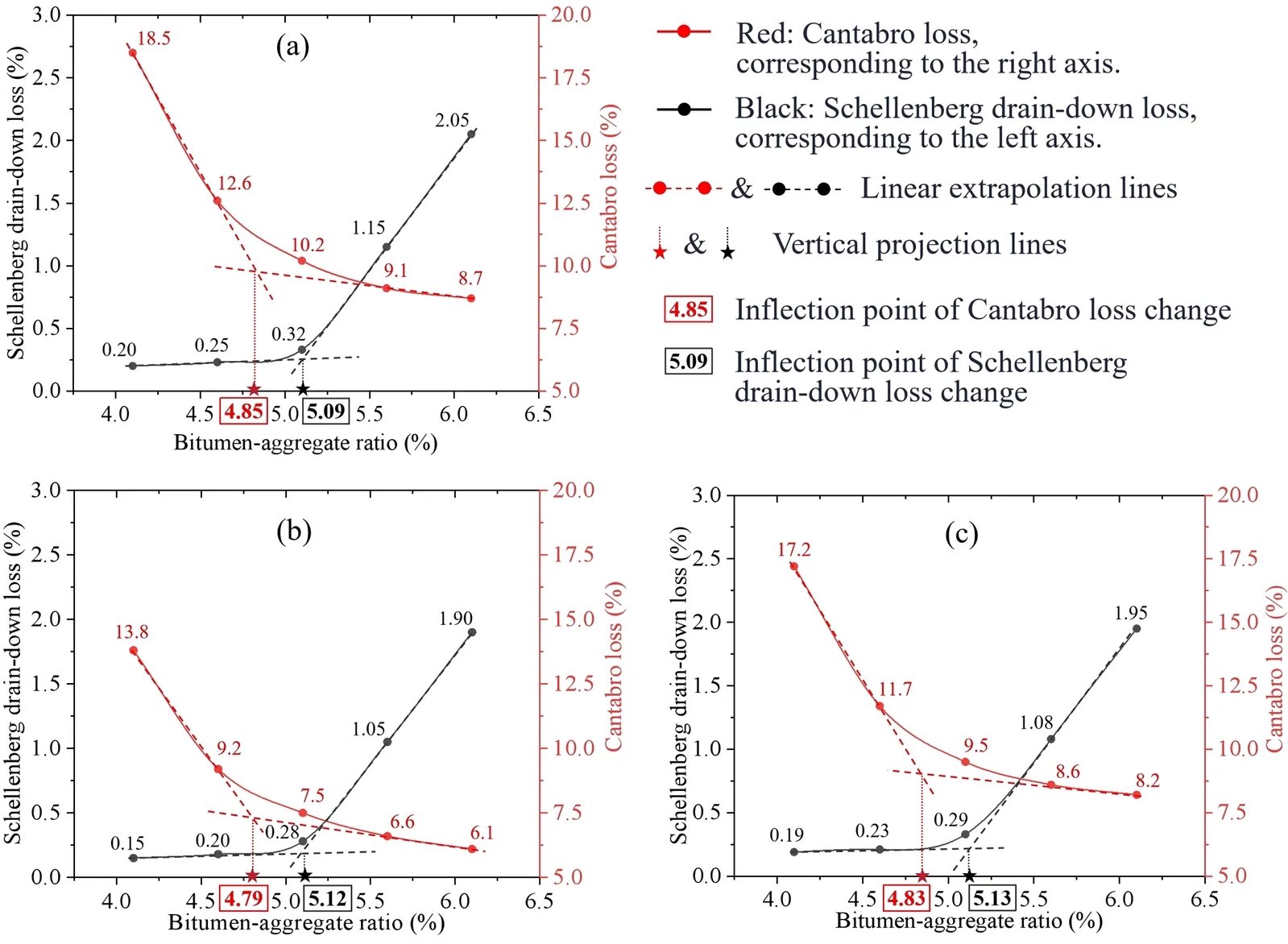
Determination of the inflection points and the optimum asphalt–aggregate ratio: (**a**) Gradation 1; (**b**) Gradation 2; (**c**) Gradation 3.

Because the three gradations have similar specific mineral aggregate surface areas and all adopt a target asphalt film thickness of 14 μm, the estimated asphalt–aggregate ratio calculated by the method described in Section 3.3 is 5.1% for all of them. Taking this value as the center, five asphalt–aggregate ratios of 4.1%, 4.6%, 5.1%, 5.6% and 6.1% were tested at intervals of 0.5%. The results are shown in Figure 8.

The test results show that the Cantabro loss of all three gradations decreases gradually as the asphalt–aggregate ratio increases, whereas the Schellenberg drain-down loss remains low, below 5.1%, and increases markedly at 5.6% and above. According to the shape of the curves in Figure 8, the asphalt–aggregate ratios controlled by raveling resistance are approximately 4.85%, 4.79% and 4.83% for Gradations 1, 2 and 3, respectively, and those controlled by drain-down resistance are approximately 5.09%, 5.12% and 5.13%, respectively. An asphalt–aggregate ratio of 5.1% therefore lies essentially within the reasonable balance range between raveling resistance and drain-down resistance for all three gradations.

Taking raveling resistance, drain-down resistance and the consistency of the subsequent between-group comparison into account, an asphalt–aggregate ratio of 5.1% was finally adopted for all three gradations. At this ratio, the Cantabro loss and the drain-down loss of all three gradations satisfy the technical requirements of JTG/T 3350-03-2020 [8], so the mixtures are suitable for the subsequent performance evaluation.

## 4. Performance Verification and Discussion

In this section, specimens were prepared using the three gradations established in Section 3 at a uniform asphalt–aggregate ratio of 5.1%. Gradations 1 and 2, which were designed using the CBR-CAVF method, were used to evaluate the target air void realization and the resulting pavement, meso-structural and acoustic performance. Gradation 3, which was established empirically, was included as a control for performance comparison rather than as a validation case for the CAVF volumetric calculation.

### 4.1. Macroscopic Pavement Performance

#### 4.1.1. Air Void Structure and Drainage Performance

The void structure is the basis on which a porous asphalt mixture develops its functional characteristics. As shown in Table 7, the measured air void contents of Gradations 1 and 2 are 21.16% and 20.64%, respectively, corresponding to deviations of 0.16 and 0.36 percentage points from the design target of 21.0%. This result indicates that the CAVF volumetric calculation, corrected by both the skeleton interference coefficient and the effective binder volume, enabled the two investigated PAC-13 design gradations to achieve air void contents close to the selected target of 21.0%. Its predictive accuracy at other target air void contents remains to be established through additional validation. The connected air void contents of the three gradations are all above 14.5%, and their permeability coefficients are all far higher than the technical requirement of 5000 mL/min, indicating satisfactory drainage performance for the mixtures tested.

Regarding the differences between the gradations, Gradation 1 has the highest connected air void content and permeability coefficient, indicating that interconnected pore channels more favorable to water flow are formed inside it. This is related to its higher proportion of the 5–10 mm fraction and the relatively balanced particle size distribution of its coarse aggregate: the skeleton formed by medium-sized particles has a more continuous pore size distribution, so the voids interconnect easily. Gradation 2, dominated by large particles and containing a larger filling volume of fine aggregate, has a slightly lower permeability coefficient, which nevertheless remains far above the technical requirement. Gradation 3 lies between the two. The ratio of the connected to the total air void content is stable at about 71% for all three gradations, which agrees with the void connectivity characteristics of PAC reported in previous studies and also indicates that the differences in drainage capacity among the three gradations arise mainly from the morphology of the pore channels rather than from the connectivity ratio itself [32].

#### 4.1.2. Mechanical Strength and High-Temperature Stability

Mechanical strength and high-temperature stability mainly reflect the ability of a PAC mixture to resist shear deformation and rutting under traffic loading and high temperature. As shown in Table 8, Gradation 2 has the highest Marshall stability and dynamic stability among the three gradations, with values exceeding those of Gradation 3 by 43.8% and 18.3%, respectively. The corresponding values for Gradation 1 exceed those of Gradation 3 by 21.9% and 6.3%, respectively. Gradation 2 also has the lowest flow value. The ranking of the dynamic stability and Marshall stability of the three gradations (Gradation 2 > Gradation 1 > Gradation 3; dynamic stability: 7384 > 6636 > 6242 passes/mm; Marshall stability: 10.5 > 8.9 > 7.3 kN) is fully consistent with the *CBR*_5.0_ ranking of their coarse aggregate skeletons (39.8% > 35.9% > 28.0%).

This correspondence agrees with the skeleton interlocking mechanism. Gradation 2 adopts a coarse aggregate ratio of 55:45, and its skeleton shows the strongest resistance to slippage and rearrangement under penetration loading. This skeleton characteristic may help maintain stable coarse aggregate contacts under high-temperature loading and thereby contribute to the higher dynamic stability of Gradation 2. Previous studies have likewise shown that porous asphalt mixtures with more complete skeleton interlock possess higher skeleton strength and better resistance to permanent deformation [13,14]. By contrast, although Gradation 3 satisfies the conventional gradation requirements, the relative proportion of its two coarse fractions (about 46.5:53.5) falls within the low-value range of the CBR test, consistent with its lowest Marshall stability and dynamic stability among the three mixtures. The mineral filler and fine aggregate contents also vary among the mixtures (Table 5) and may contribute to the measured performance differences. Accordingly, the mechanical performance comparison is interpreted in relation to both skeleton interlock and the accompanying compositional differences. Together, these observations support the use of *CBR*_5.0_ as a mechanical basis for coarse aggregate screening within the CBR-CAVF procedure, followed by volumetric proportioning and mixture performance verification.

#### 4.1.3. Raveling Resistance and Moisture Stability

A PAC mixture serves for a long time under the combined action of large air voids and a wet environment, so raveling resistance and moisture stability are important indices of its structural durability. As shown in Table 9, the binder drain-down loss, Cantabro loss, water-immersed Cantabro loss, residual Marshall stability after immersion and freeze–thaw indirect tensile strength ratio of the three gradations all satisfy the technical requirements, indicating that, at the uniform asphalt–aggregate ratio of 5.1%, all three mixtures possess basic binder retention capacity and resistance to moisture damage.

Regarding the differences between the gradations, Gradation 2 shows better overall raveling resistance and moisture stability. On the one hand, this is related to the higher interlocking strength of its coarse aggregate skeleton: a stable coarse aggregate contact structure reduces the risk of particle slippage, rearrangement and loosening under loading, which agrees with the influence of the degree of skeleton interlock on the strength and durability of porous asphalt mixtures reported in previous studies [13,14]. On the other hand, the larger fine aggregate volume in Gradation 2 helps to improve the filling state of the mastic within the skeleton voids and to enhance the bonding stability between aggregates. Gradation 1 has a higher connected air void content and permeability coefficient and thus a more prominent drainage function, but its raveling resistance and moisture stability are slightly lower than those of Gradation 2, indicating that a more open pore structure, while improving the drainage capacity, may increase the risks of water entering the interior of the mixture and of particle loosening.

### 4.2. Meso-Scale Skeleton Structure

**Figure 9:**
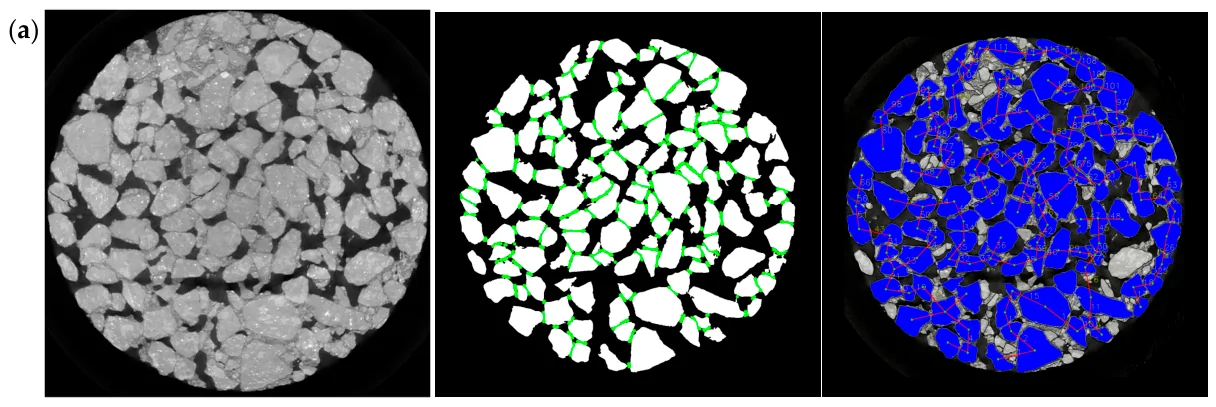

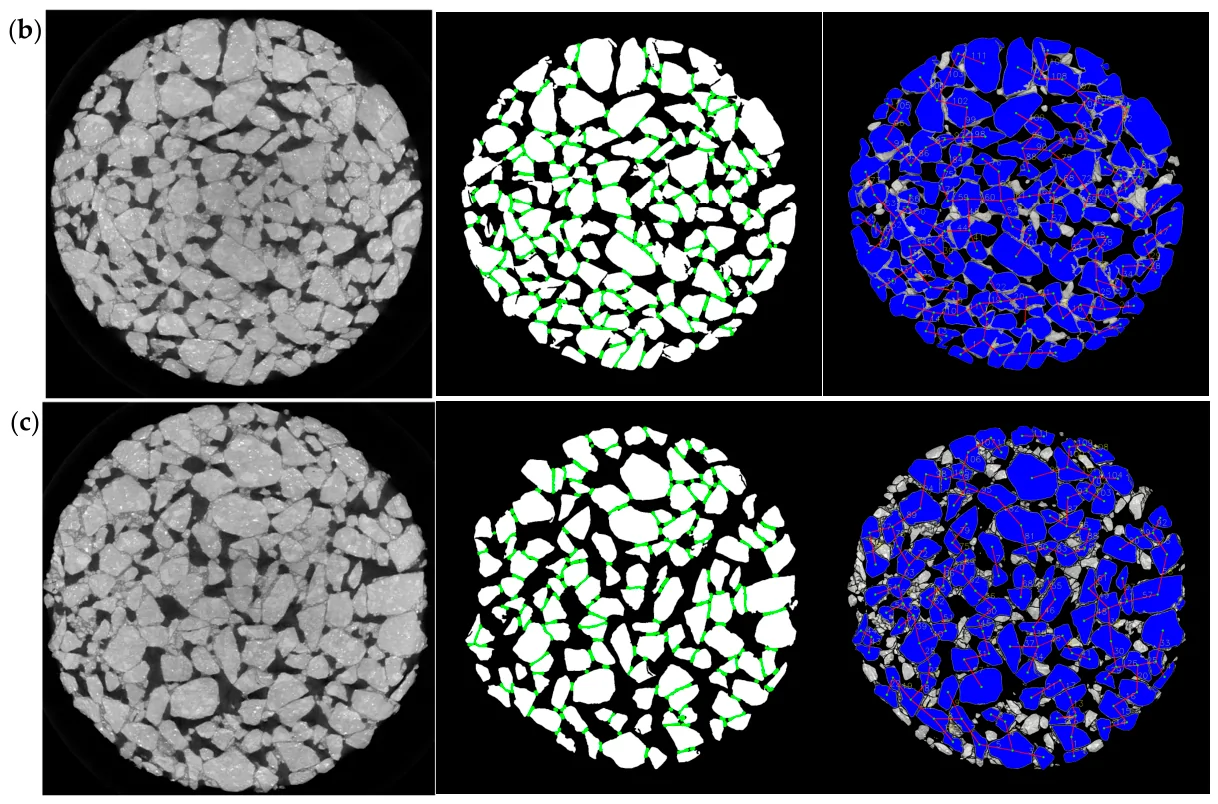
Skeleton analysis images of cross-sections at a depth of 30 mm for specimens with three types of gradation: (**a**) Gradation 1; (**b**) Gradation 2; (**c**) Gradation 3.

**Figure 10:**
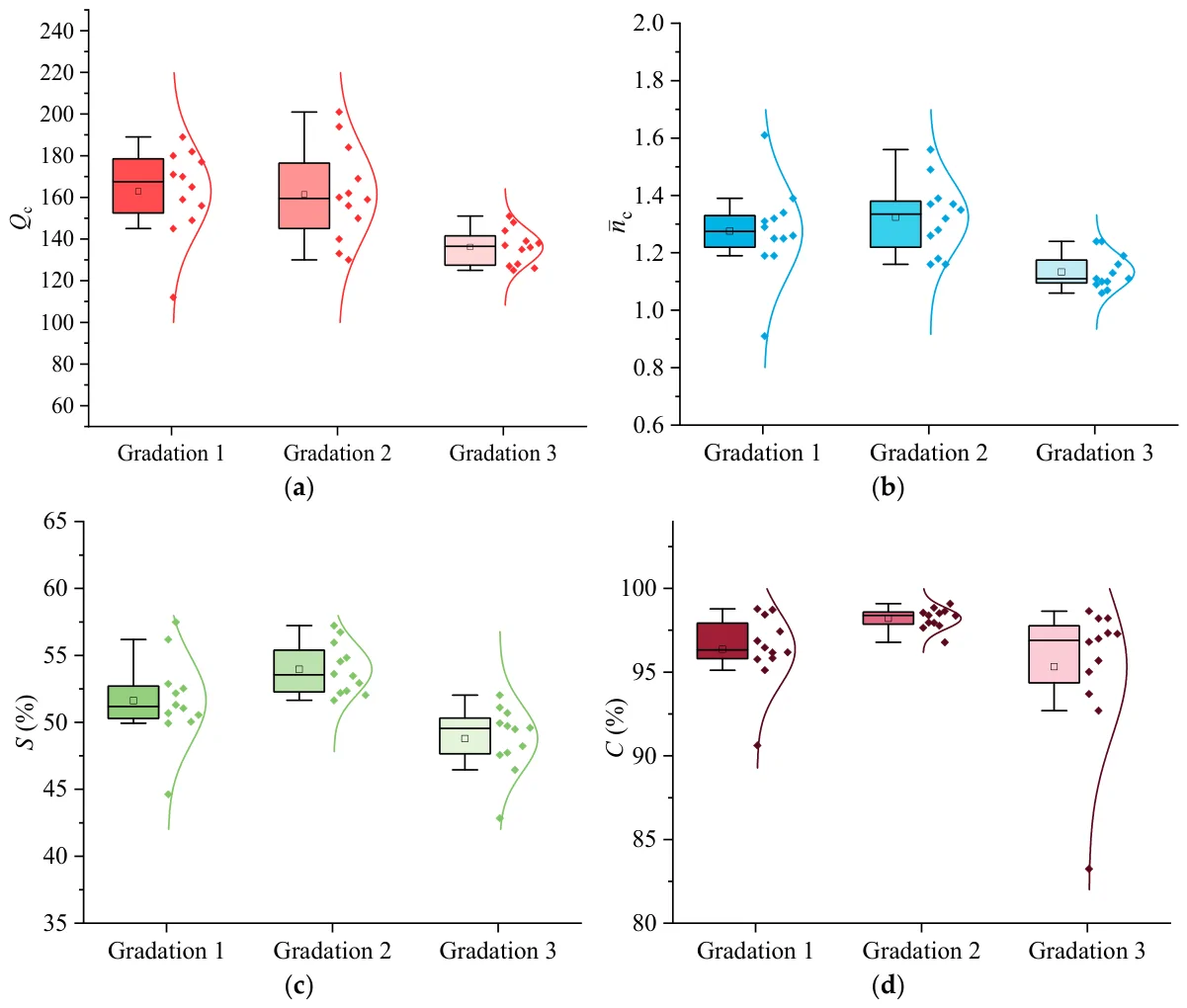
Distributions of the skeleton test results for the different gradations: (**a**) number of contact points; (**b**) mean coordination number; (**c**) skeleton ratio; (**d**) aggregate contact ratio.

Following the method described in Section 2.5.2, representative specimens of the three gradations were scanned by CT and subjected to multi-section statistical analysis of the skeleton. Examples of the identified skeleton images of the CT cross-sections of the three gradations are shown in Figure 9, and the multi-section statistical results are given in Figure 10 and Table 10. As shown in Table 10, the numbers of contact points *Q*_c_ of Gradations 1 and 2 are about 19.7% and 18.7% higher than that of Gradation 3, respectively, indicating that the two gradations designed by the CBR-CAVF method form richer coarse aggregate contact relationships after compaction. However, *Q*_c_ is strongly affected by the number of coarse aggregate particles within a cross-section; it reflects only the total number of contacts and cannot by itself evaluate the quality of the skeleton contact network. *Q*_c_ of Gradation 1 is only 0.9% higher than that of Gradation 2, whereas its ${\bar{n}}_{c}$, *S* and *C* are all lower than those of Gradation 2, indicating that a larger number of contact points does not necessarily correspond to a more efficient load-bearing skeleton.

The mean coordination number, the skeleton ratio and the aggregate contact ratio further reveal the skeleton differences among the three gradations. The mean coordination number ${\bar{n}}_{c}$ of Gradation 2 is 3.1% and 16.8% higher than those of Gradations 1 and 3, respectively, indicating that each of its coarse aggregate particles is, on average, more fully in contact with its neighbors and that the interlocking and confinement between particles are stronger. Meanwhile, the skeleton ratio *S* of Gradation 2 is 4.5% and 10.6% higher than those of Gradations 1 and 3, respectively, indicating that the coarse aggregate area participating in the effective skeleton accounts for a larger share of the whole cross-section. The aggregate contact ratio *C* of Gradation 2 is 1.9% and 3.0% higher than those of Gradations 1 and 3, respectively, indicating that the proportion of its coarse aggregate participating in the effective contact network is the highest. By contrast, the *Q*_c_, ${\bar{n}}_{c}$ and *S* of Gradation 3 are all at relatively low levels, indicating that, although the conventional empirical gradation satisfies the usual sieve passing and target air void requirements, its coarse aggregate contact and interlocking structure is not fully developed.

The multi-section CT statistics provide particle-scale evidence from the scanned specimens for the CBR-based optimization of the coarse aggregate. The mean coordination number, the skeleton ratio *S* and the aggregate contact ratio *C* all follow the order Gradation 2 > Gradation 1 > Gradation 3, consistent with the rankings of the skeleton CBR5.0 and of the Marshall stability and dynamic stability of the mixtures. A higher coordination number may allow the load on a particle to be distributed over more contact points, reducing the tendency for particle slippage and rearrangement. This structural interpretation is consistent with the higher dynamic stability of Gradation 2. The *Q*_c_ of Gradation 1 is slightly higher than that of Gradation 2, while all its other indices are lower, suggesting that this gradation tends to form numerous but relatively dispersed local contacts. The number of contacts is not equivalent to the quality of the skeleton, and skeleton evaluation should focus on the sufficiency and continuity of the contacts, in agreement with the conclusions of previous meso-scale studies [33,34]. These results support, at the meso scale, the selection of the coarse aggregate skeleton within the CBR-CAVF method.

Although Gradation 3 satisfies the conventional PAC-13 gradation and total air void requirements, its coarse fraction ratio of approximately 46.5:53.5 is close to the low-value range identified by CBR screening, and its skeleton indices and mechanical performance are the lowest among the three gradations. This comparison supports using mechanical skeleton screening to complement sieve-based gradation control. In addition, Gradations 1 and 3 have lower skeleton indices at a depth of 5 mm than in the interior, suggesting a near-surface compaction boundary effect.

### 4.3. Sound Absorption Performance

Using the procedure described in Section 2.5.3, the three PAC-13 gradations were compared with AC-13 and SMA-13 reference mixtures, which used the median gradations specified in JTG F40-2004 [31] and asphalt–aggregate ratios of 4.8% and 6.0%, respectively. All specimens were prepared by SGC with 50 gyrations, had a diameter of 100 mm and a thickness of 63.5 ± 1.3 mm and were tested under the same normal-incidence, rigid-backed conditions. Two specimens per gradation were tested at three circumferential orientations each, and the six measurements were averaged at each frequency.

**Figure 11:**
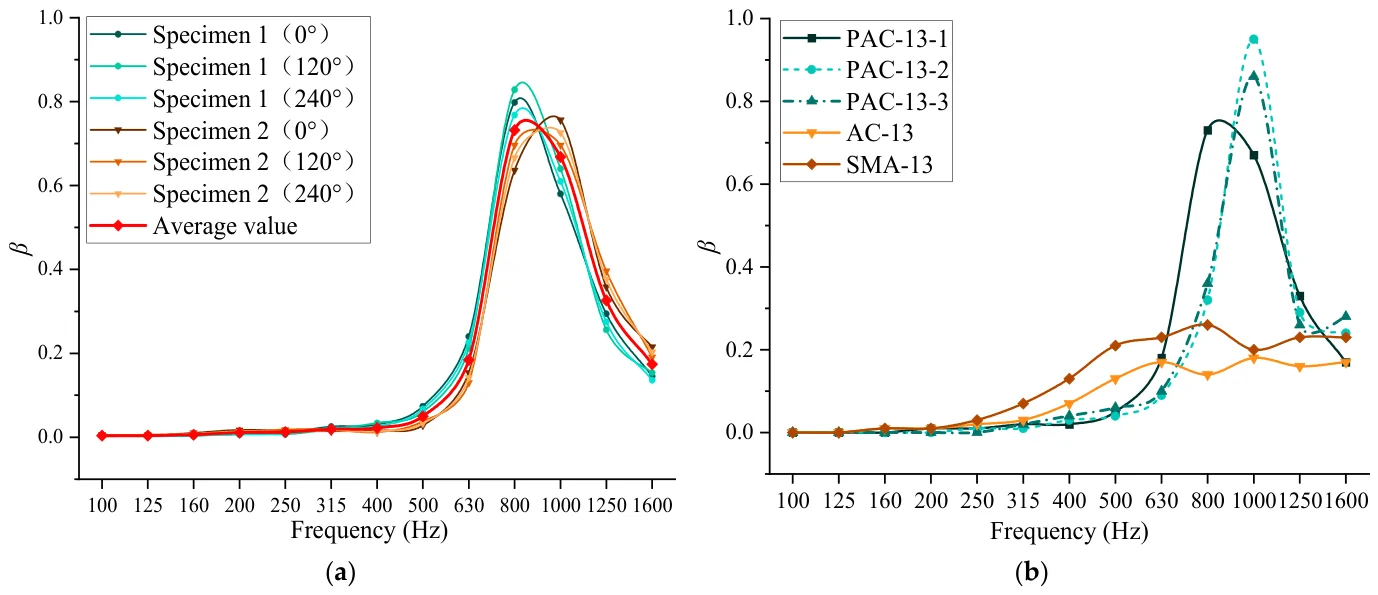
Sound absorption coefficient test curve for the mixture: (**a**) sound absorption coefficient of Gradation 1 as a function of frequency; (**b**) sound absorption coefficients of the different mixtures as a function of frequency.

Taking Gradation 1 as an example, its six sets of results are summarized in Figure 11a. The overall trends of the six data sets are consistent: the sound absorption coefficients at 800 Hz and 1000 Hz lie in the ranges of 0.713–0.748 and 0.654–0.685, respectively, and the absorption peak remains near 800 Hz in every set, indicating that the rotation tests had good consistency.

Figure 11b shows low absorption at 100–400 Hz and increased absorption for the PAC-13 mixtures above 500 Hz. The 500–1600 Hz band used for $\bar{\beta }$ covers frequencies relevant to tire–pavement noise [44]. The sound absorption indices of the five mixtures are compared in Table 11.

Under the adopted laboratory conditions, the mean normal-incidence absorption coefficients of the PAC-13 mixtures are 2.0–2.3 times those of AC-13 and 1.4–1.6 times those of SMA-13. The different responses among the three PAC-13 gradations, despite their similar total air void contents, are consistent with the influence of pore connectivity, morphology and tortuosity on acoustic energy dissipation [35,36].

Gradation 1 has the highest $\bar{\beta }$ (0.356), with *β*_max_ = 0.732 at 800 Hz, indicating the strongest average absorption over 500–1600 Hz among the three PAC gradations. This is consistent with its higher connected air void content and permeability. Gradation 2 reaches the highest *β*_max_ at 1000 Hz, but its $\bar{\beta }$ is 0.322, indicating a more pronounced peak response. Gradation 3 also peaks at 1000 Hz, with *β*_max_ = 0.859 and $\bar{\beta }$ = 0.320.

### 4.4. Applicability and Limitations

The present results support the feasibility of the CBR-CAVF procedure for the investigated PAC-13 mixtures. The close agreement between the target and measured air void contents of the two design gradations, together with the consistent rankings of CBR5.0, the principal CT skeleton indices and mechanical performance, provides complementary evidence under the adopted experimental conditions. However, the validation was limited to one material system, the selected compaction conditions and a target air void content of 21.0%. The selected mineral filler contents, including the 1.5% used for Gradation 2, were treated as input assumptions in the volumetric design. *α* = 1.120 was calibrated using Gradation 3, and its application to the two design gradations represents an approximation within this material system. The performance comparisons concern the combined effects of skeleton selection and constituent proportioning. The applicability of the procedure to other aggregate sources, binder systems, nominal maximum aggregate sizes and target air void contents remains to be established. The selected skeleton proportions and the calibrated interference coefficient should therefore be re-evaluated when these conditions change.

With one specimen scanned per gradation, the CT results provide descriptive evidence of the contact structure and its variation among sections within each specimen. The sound absorption results characterize normal-incidence behavior under the tested specimen geometry and rigid-backed boundary condition; their implications for field noise reduction remain to be evaluated. Further validation across a wider range of materials and design targets is needed to establish the broader applicability of the proposed procedure.

## 5. Conclusions

In this study, a design method for porous asphalt mixtures based on CBR optimization of the coarse aggregate skeleton and a modified CAVF volumetric design was proposed and evaluated using PAC-13 mixtures through macroscopic pavement performance, CT meso-structure and sound absorption tests. The following conclusions apply to the material system, compaction conditions and target air void content investigated in this study:

(1) The modified CBR penetration test can quantitatively optimize the coarse aggregate skeleton proportion at the front end of the design. *CBR*_5.0_ fluctuates non-monotonically with the proportion of the 10–15 mm fraction, revealing several locally favorable packing states. On this basis, the two coarse aggregate combinations with 10–15 mm and 5–10 mm at mass ratios of 40:60 and 55:45 were selected, with mean CBR5.0 values of 35.9% and 39.8%, respectively, turning the determination of the skeleton proportion from empirical judgment into control by a mechanical index.
(2) For the investigated PAC-13 mixtures, measuring the voids in the coarse aggregate skeleton by SGC and applying the double correction of the skeleton interference coefficient (α = 1.120) and the effective binder volume enabled the two CBR-CAVF design gradations to achieve air void contents close to the target of 21.0%. The measured air void contents were 21.16% and 20.64%, corresponding to deviations of 0.16 and 0.36 percentage points, respectively. These results support the volumetric design accuracy under the investigated conditions. The drainage performance of all three gradations satisfied the technical requirements.
(3) The observed rankings show agreement between the skeleton CBR5.0, the principal meso-scale contact indices and the measured mechanical performance. The ranking of the Marshall stability and dynamic stability of the three gradations (Gradation 2 > Gradation 1 > Gradation 3; Marshall stability: 10.5 > 8.9 > 7.3 kN; dynamic stability: 7384 > 6636 > 6242 passes/mm) coincides with the rankings of the skeleton *CBR*_5.0_ (39.8% > 35.9% > 28.0%) and of the principal CT meso-scale indices. In the scanned specimens, the mean coordination numbers were 1.32, 1.28 and 1.13 for Gradations 2, 1 and 3, respectively. The Marshall stability and dynamic stability of Gradation 2 are 43.8% and 18.3% higher than those of the control gradation, respectively. These results support the value of combining CBR-based skeleton screening with volumetric proportioning in the design of the investigated PAC-13 mixtures.
(4) The results indicate the potential of the CBR-CAVF method to support function-oriented design for the investigated PAC-13 mixtures. The mean normal-incidence sound absorption coefficients of the three PAC gradations over the 500–1600 Hz band range from 0.320 to 0.356 and are 2.0–2.3 times that of AC-13 and 1.4–1.6 times that of SMA-13. At the same target air void content, the two designed gradations exhibit different performance emphases: Gradation 1 has the highest permeability and mean sound absorption coefficient (7920 mL/min and 0.356, respectively), indicating potential benefits for sections prioritizing drainage and noise reduction, whereas Gradation 2 has the best overall mechanical and durability-related performance among the tested PAC gradations, suggesting potential suitability for heavy-load and high-temperature conditions.

## Figures and Tables

**Table 1:** Main properties of high-viscosity modified asphalt and granular lignin fiber.

High-viscosity modified asphalt	Penetration at 25 °C (0.1 mm)	Softening point (ring-and-ball) (°C)	Ductility at 5 °C (cm)
	47	91	31
	Dynamic viscosity at 60 °C (Pa·s)	Brookfield rotational viscosity at 170 °C (Pa·s)	Penetration ratio after RTFOT (%)
	401,388	0.98	86.1
Lignin fiber (granular)	Density (g/cm^3^)	Oil absorption ratio	Mean fiber length (mm)
	1.080	6.5	0.5

**Table 2:** Main properties of the mineral aggregates and filler.

Coarse aggregate	Crushed value (%)	Los Angeles abrasion loss (%)	Apparent relative density	Flat and elongated particle content (%)
	10.6	11.7	2.940 (10–15 mm); 2.930 (5–10 mm)	4.6
Fine aggregate	Sand equivalent (%)	Angularity (s)	Apparent relative density	Soundness (>0.3 mm fraction) (%)
	72	37.9	2.921	2.8
Filler	Apparent relative density of mineral filler	Hydrophilic coefficient of mineral filler	Plasticity index of mineral filler (%)	Apparent relative density of cement
	2.716	0.7	3	3.100

**Table 3:** *VCA*_SGC_ test results of the two selected coarse aggregate combinations.

Mass Proportion of the 10–15 mm Fraction/%	40					55				
Test No.	1	2	3	4	5	1	2	3	4	5
*VCA *_SGC _(%)	36.11	36.32	36.24	36.34	36.14	37.07	36.97	37.15	37.20	37.16
Mean (%)	36.23 ± 0.10					37.11 ± 0.09				

**Table 4:** Volumetric proportions of the constituent materials of the mixtures (%).

Volumetric Proportion	10–15 mm Aggregate	5–10 mm Aggregate	0–3 mm Aggregate	Mineral Filler	Cement	Fiber	Estimated Effective Asphalt	Air Voids
Gradation 1	23.72	35.70	7.58	1.50	0.65	0.60	9.25	21.00
Gradation 2	32.09	26.34	8.95	1.12	0.65	0.60	9.25	21.00

**Table 5:** Mass composition of the mineral aggregate for the three gradations (%).

Mass Proportion	10–15 mm Aggregate	5–10 mm Aggregate	0–3 mm Aggregate	Mineral Filler	Cement
Gradation 1 (calculated)	34.40	51.62	10.98	2.00	1.00
Gradation 1 (rounded)	34.50	51.50	11.00	2.00	1.00
Gradation 2 (calculated)	46.54	38.07	12.89	1.50	1.00
Gradation 2 (rounded)	46.50	38.00	13.00	1.50	1.00
Gradation 3 (control)	40.00	46.00	11.00	2.00	1.00

**Table 6:** Comparison of the percentages passing (%) for the three gradations.

Sieve Size (mm)	16	13.2	9.5	4.75	2.36	1.18	0.6	0.3	0.15	0.075
Gradation 1	100.0	96.7	65.2	16.1	13.2	11.1	8.7	6.6	5.3	4.2
Gradation 2	100.0	95.5	55.1	17.3	14.6	12.1	9.2	6.8	5.3	4.1
Gradation 3	100.0	96.1	60.5	16.0	13.3	11.1	8.7	6.7	5.3	4.3

**Table 7:** Comparison of the drainage capacity indices of the mixtures with different gradations.

Test Index	Unit	Criteria	Gradation 1	Gradation 2	Gradation 3
Air void content (*n* = 5)	%	18–25	21.16 ± 0.17	20.64 ± 0.23	20.80 ± 0.35
Connected air void content (*n* = 5)	%	—	14.96 ± 0.18	14.59 ± 0.23	14.77 ± 0.19
Water permeability (*n* = 3)	mL/min	≥5000	7920 ± 278	7310 ± 189	7586 ± 113

**Table 8:** Comparison of the mechanical strength and high-temperature stability indices of the mixtures with different gradations.

Test Index	Unit	Criteria	Gradation 1	Gradation 2	Gradation 3
Skeleton *CBR*_5.0_ (*n* = 3)	%	—	35.9 ± 2.6	39.8 ± 3.1	28.0 ± 2.2
Marshall stability (*n* = 5)	kN	≥5.0	8.9 ± 0.5	10.5 ± 0.5	7.3 ± 0.3
Flow value (*n* = 5)	mm	—	3.2 ± 0.2	2.8 ± 0.2	3.5 ± 0.3
Dynamic stability (60 °C) (*n* = 3)	passes/mm	≥5000	6636 ± 274	7384 ± 388	6242 ± 310

**Table 9:** Comparison of durability-related performance indices of mixtures with different gradations.

Test Index	Unit	Criteria	Gradation 1	Gradation 2	Gradation 3
Schellenberg drain-down loss (*n* = 3)	%	≤0.8	0.32 ± 0.03	0.28 ± 0.01	0.29 ± 0.03
Cantabro loss (*n* = 4)	%	≤15	10.2 ± 0.6	7.5 ± 0.2	9.5 ± 0.7
Water-immersed Cantabro loss (*n* = 4)	%	≤20	13.7 ± 0.4	11.8 ± 0.7	13.3 ± 0.8
Residual Marshall stability after immersion (*n* = 4)	%	≥85	89.1 ± 2.0	91.5 ± 1.7	90.3 ± 1.1
Freeze–thaw indirect tensile strength ratio (*n* = 4)	%	≥80	86.7 ± 1.4	88.1 ± 0.6	87.7 ± 1.5

**Table 10:** Statistical results of the skeleton analysis.

Test Index	Statistic	Gradation 1	Gradation 2	Gradation 3
Number of contact points, $Q_{c}$	Mean ± SD	162.9 ± 20.94	161.5 ± 22.60	136.1 ± 8.60
	CV (%)	12.9	14.0	6.3
Mean coordination number, ${\bar{n}}_{c}$	Mean ± SD	1.28 ± 0.16	1.32 ± 0.13	1.13 ± 0.06
	CV (%)	12.6	9.8	5.5
Skeleton ratio, S (%)	Mean ± SD	51.62 ± 3.23	53.96 ± 1.89	48.78 ± 2.46
	CV (%)	6.3	3.5	5.0
Aggregate contact ratio, C (%)	Mean ± SD	96.37 ± 2.18	98.21 ± 0.63	95.32 ± 4.23
	CV (%)	2.3	0.6	4.4

Note: SD denotes standard deviation; CV denotes the coefficient of variation and is expressed as a percentage. For each gradation, the mean, standard deviation and coefficient of variation were calculated from 12 cross-sections of one CT-scanned specimen and describe variation among sections within that specimen.

**Table 11:** Sound absorption test results of the different mixtures.

Mixture Type	Peak Sound Absorption Coefficient *β*_max_	Peak Frequency *f*_p_ (Hz)	Mean Sound Absorption Coefficient $\bar{\beta }$ over 500–1600 Hz
PAC-13 Gradation 1	0.732 ± 0.013	800	0.356 ± 0.004
PAC-13 Gradation 2	0.949 ± 0.029	1000	0.322 ± 0.005
PAC-13 Gradation 3	0.859 ± 0.042	1000	0.320 ± 0.005
AC-13	0.177 ± 0.017	1000	0.158 ± 0.002
SMA-13	0.261 ± 0.015	800	0.227 ± 0.004

Note: Values are reported as the mean ± SD across six measurement sets obtained from two specimens tested at three orientations each. The SD describes variation across these measurement sets and does not represent six independent specimens.

## Data Availability

The data presented in this study are available within the article. Additional data are available from the corresponding author upon reasonable request.

## Nomenclature

Symbol	Definition	Unit
${CBR}_{L}$, ${CBR}_{5.0}$	California Bearing Ratios at penetration depths of L mm and 5.0 mm, respectively	%
L	Penetration depth	mm
$F_{L}$	Measured penetration load at depth L	N
$P_{L}$	Standard penetration pressure at depth L	MPa
* A *_p_	Cross-sectional area of the penetration piston	mm^2^
${VCA}_{\mathrm{SGC}}$	Voids in the coarse aggregate skeleton compacted alone by Superpave gyratory compaction	%
${VCA}_{\mathrm{DRC}}$	Voids in the dry-rodded coarse aggregate skeleton	%
${VCA}_{\mathrm{mix}}$	Voids in coarse aggregate within the compacted asphalt mixture	%
α	Skeleton interference coefficient, defined as ${VCA}_{mix}/{VCA}_{\mathrm{SGC}}$	–
$\rho_{\mathrm{SGC}}$ **,** $\rho_{\mathrm{DRC}}$	Bulk densities of the coarse aggregate skeleton after gyratory compaction and dry rodding, respectively	g/cm^3^
$\rho_{w}$	Density of water	g/cm^3^
$\gamma_{\mathrm{cab}}$	Combined bulk specific gravity of the coarse aggregate blend on a dry basis	–
$\gamma_{\mathrm{mb}}$	Bulk specific gravity of the compacted asphalt mixture	–
$\gamma_{f}$ **,** $\gamma_{p}$	Apparent relative densities of fine aggregate and filler, respectively	–
$m_{\mathrm{ca}}$	Mass of the coarse aggregate blend used for skeleton compaction	g
$A_{m}$	Internal cross-sectional area of the compaction mold	cm^2^
$h_{\mathrm{ca}}$	Mean height of the compacted coarse aggregate skeleton	cm
$q_{c}$ **,** $q_{f}$ **,** $q_{p}$	Mass percentages of coarse aggregate, fine aggregate and filler in the total mineral aggregate, respectively	%
$P_{\mathrm{ca}}$	Mass percentage of coarse aggregate in the total asphalt mixture	%
$V_{v}$	Target air void content of the asphalt mixture	%
$V_{c}$	Connected air void content of the asphalt mixture	%
$V_{b}$ **,** $V_{\mathrm{be}}$	Total and effective binder volume percentages in the mixture, respectively	%
$V_{f}$	Volume percentage of fine aggregate in the mixture	%
$V_{p}$	Combined volume percentage of filler (including cement) and fiber in the mixture	%
$V_{m}$ **,** $V_{t}$	Total specimen volume and specimen volume excluding connected voids, respectively	cm^3^
$m_{d}$ **,** $m_{w}$	Dry specimen mass and apparent specimen mass when weighed in water, respectively	g
$Q_{c}$	Total number of contacts between coarse aggregate particles in a cross-section	–
${\bar{n}}_{c}$	Mean coordination number of coarse aggregate particles in a cross-section	–
N **,** $n_{i}$	Number of coarse aggregate particles in a cross-section and number of contacts of particle i, respectively	–
S	Skeleton ratio: area of coarse aggregate participating in the effective skeleton divided by the specimen cross-sectional area	%
C	Aggregate contact ratio: area of coarse aggregate participating in the effective skeleton divided by the total coarse aggregate area	%
$A_{t}$ **,** $A_{s}$ **,** $A_{c}$	Specimen cross-sectional area, area of coarse aggregate participating in the effective skeleton and total coarse aggregate area, respectively	mm^2^
β **,** $\beta_{i}$	Normal-incidence sound absorption coefficient, with subscript i denoting the test frequency	–
$\bar{\beta }$	Arithmetic mean of the sound absorption coefficients at the test frequencies within 500–1600 Hz	–
$\beta_{\max}$	Maximum sound absorption coefficient among the test frequencies	–
$f_{p}$	Frequency corresponding to the maximum sound absorption coefficient	Hz
$S_{i}$	Standing wave ratio, with subscript i denoting the test frequency	–
$p_{\max}$ **,** $p_{\min}$ **;** $p_{\max,i}$ **,** $p_{\min,i}$	Maximum and minimum sound pressure amplitudes; subscript i denotes the test frequency	Pa
n	Number of replicate results used for a reported statistic, as specified for each test	–
